# Electronic informed consent: effects on enrolment, practical and economic benefits, challenges, and drawbacks—a systematic review of studies within randomized controlled trials

**DOI:** 10.1186/s13063-022-06959-6

**Published:** 2023-02-21

**Authors:** Ana Teresita Mazzochi, Martin Dennis, Ho-Yan Yvonne Chun

**Affiliations:** 1 Usher Institute, University of Edinburgh, Old Medical School, Teviot Place, Edinburgh, EH8 9AG UK; 2 Centre for Clinical Brain Sciences, University of Edinburgh, Edinburgh, UK

**Keywords:** Electronic informed consent, Enrollment, SWAT, Randomized controlled trials, Systematic review

## Abstract

**Background:**

Enrolment is one of the most challenging aspects of conducting clinical trials, preceded by the process of informed consent (IC). Different strategies to improve recruitment in clinical trials have been used, including electronic IC. During COVID-19 pandemic, barriers to enrolment have been evident. Although digital technologies were acknowledged as the future of clinical research and potential advantages were shown for recruitment, electronic informed consent (e-IC) has not yet been globally adopted. The purpose of this review is to investigate the effect of using e-IC on enrolment, practical and economic benefits, challenges, and drawbacks when compared to traditional informed consent, through a systematic review.

**Methods:**

Embase, Global Health Library, Medline, and The Cochrane Library databases were searched. No limit was set for publication date, age, sex, or study design. We included all studies within a randomized controlled trial (RCT), published in English, Chinese or Spanish, evaluating the electronic consent process used in the parent RCT. Studies were included if any of the three components ((i) information provision, (ii) participant’s comprehension, (iii) signature) of the IC process was designed as electronic, whether administered remotely or face-to-face. The primary outcome was the rate of enrolment to the parent trial. Secondary outcomes were summarized according to the various findings reported on the use of electronic consent.

**Results:**

From a total of 9069 titles, 12 studies were included in the final analysis with a total of 8864 participants. Five studies of high heterogeneity and risk of bias showed mixed results on the efficacy of e-IC on enrolment. Data of included studies suggested e-IC could improve comprehension and recall of study-related information. Meta-analysis could not be conducted due to different study designs and outcome measures and the predominantly qualitative findings.

**Conclusion:**

Few published studies have investigated the impact of e-IC on enrolment and findings were mixed. e-IC may improve participant’s comprehension and recall of information. High-quality studies are needed to evaluate the potential benefit of e-IC to increase clinical trial enrolment.

**Trial registration:**

PROSPERO CRD42021231035. Registration date: 19-Feb-2021.

**Supplementary Information:**

The online version contains supplementary material available at 10.1186/s13063-022-06959-6.

## Introduction

Enrolment is known to be one of the most challenging aspects of conducting clinical trials [1–3]. Enrolment is preceded by the process of informed consent (IC), during which an effective communication of trial information is crucial before obtaining a participant’s IC on trial participation [4].

IC is the first trial process to ensure that potential participants are duly informed of the trial involvement and that their decision to participate is voluntary and should be free of undue influence, incentive or coercion [5]. Large-scale societal lockdowns as a response to the severe acute respiratory syndrome corona virus 2 (SARS-CoV-2) pandemic in 2020 directly impacted on the execution of clinical trials due to restrictions imposed on in-person visits. The halt in trial activities in turn led to an increased uptake in the use of digital health technologies as a viable solution for consenting and recruiting trial participants. While some aspects of electronic informed consent (e-IC) have been researched and tested in fully remote trials pre-pandemic [6], there could be a stronger demand for further evaluation of e-IC as the global health emergency has brought attention to decentralized or remote clinical trial methods (e.g. web-based trials) as potential approaches for conducting clinical research.

### Enrolment

Enrolment can be defined as a person’s agreement to participate in a clinical trial. The person’s decision on whether to take part in a clinical trial or not has underlying implications on the validity of a trial. Sample size recruited should provide sufficient statistical power in the trial data to enable precise measurement of study endpoints. Under-recruitment jeopardizes the internal validity of the trial with imprecise results. Evidence has shown that less than one-third of trials achieve their original planned sample size in time [7]. Sample recruited to a trial needs to be representative of the target population for its results to be externally valid. Enrolment methods need to minimize the degree of selection bias. Geographical location, disability of potential participants and complexity of the IC process are example barriers to accessing clinical research opportunities.

### Electronic informed consent

E-IC is defined as “the use of any electronic media (such as text, graphics, audio, video, podcasts or websites) to convey information related to the study and to seek and/or document IC via an electronic device such as a smartphone, tablet or computer” ([8], pp. 4). Potential benefits in using e-IC when compared to using paper consent include improved information provision with multimedia content and improved access to research, removing the need for travel for potential participants or research staff, which could ultimately enhance trial recruitment [9]. Potential drawbacks include the difficulty in determining a person’s capacity or if the consent was informed or voluntary, the risk of identity theft and issues around data confidentiality ([10], pp. 218) [11, 12]. IC is a complex process that can be divided into three components: (i) information provision, (ii) a participant’s comprehension assessment and (iii) obtaining a valid signature [13].

#### Information provision

Fully disclosing all the elements contemplated in ICH-GCP ([14] pp. 24) can be cumbersome and it is linked to the training, knowledge and ability of the clinical researcher to express difficult concepts in understandable words in order to ensure that the person is fully informed to take a free and voluntary decision. Too much information may be detrimental to the person’s understanding while others may consider it a breach of person’s rights when too little information is provided [15, 16].

#### Participant comprehension assessment

Comprehension can be affected by a number of different factors such as the following: the capacity of the researcher to effectively communicate with the potential participant, the amount of time dedicated to the process of providing information and assessing comprehension, the level of literacy of the potential participant and/or legal representative, the health condition of the potential participant that may reduce their capacity to understand and the readability and/or layout of their document [17]. The assessment of information comprehension may become a challenge when the IC process is carried out remotely. Methods should be in place to ensure potential participant has adequate understanding of the information given before consenting to the trial.

#### Obtaining a valid signature

ICH-GCP guidelines state that both the potential participant/legally acceptable representative (LAR) and the person who conducted the IC discussion should sign and personally date the written IC form ([14], Sect. 4.8.8). The introduction of web technologies in clinical research has brought about the possibility of replacing wet-ink signature with electronic signature. The U.S. Food and Drug Administration (FDA) [18] guidance considers electronic signature equivalent to full handwritten signature when it complies with the Code of Federal Regulations ([18] pp. 7). In the UK, the Joint Statement on Seeking Consent by Electronic Methods distinguishes between different types of electronic signature and considers different scenarios when deciding which type of signature is best to adopt [8]. Privacy and data protection concerns have limited the use of electronic signature for IC in other countries [19].

### Rationale for this review

#### Evidence before this review

The PRioRiTy trial, a study which identified research priorities for how to improve the process of recruitment and retention in RCT [20], has acknowledged IC optimization as an area that requires further research to improve enrolment. While systematic reviews on strategies to improve recruitment to randomized trials have been conducted, they did not focus on e-IC as the intervention [21, 22]. Other reviews that analysed the impact of digital tools on recruitment were not related to the process of IC [23, 24] or were solely aimed at the first component (information provision) of the IC process being administered electronically [25, 26].

#### Potential impact

Regulatory agencies and various private–public partnerships [27–29] have acknowledged the vital role played by digital technologies in the future of clinical research, recognizing the potential advantages they bring to recruitment and process quality. In spite of this, e-IC has not yet been globally adopted [30]. A systematic review is necessary to summarize the latest evidence on the process of e-IC as a key step in improving the process of IC and enrolment to clinical research.

### Aim

The overarching aim is to investigate the effect of e-IC on enrolment, practical and economic benefits, challenges and drawbacks of using e-IC when compared to traditional IC, through a systematic review.

### Research questions

1. Does the use e-IC (any of the three components) improve enrolment rate: proportion of invited potential participants enrolled and/or number of participants recruited in a given period (e.g. month)?
  The three components of the consent process are as follows: (i) information provision, (ii) assessment of participant’s comprehension and (iii) the signature process.
2. To summarize available research findings, including qualitative information on the use of e-IC (any of the three components): the practical and economic benefits and challenges, drawbacks, acceptability by patients, feasibility, e.g. failure to complete consent process thus needing to switch over to paper consent, and other findings that the author may find relevant during this review.

## Methods

### Ethical considerations

This project was not submitted for considerations by research ethics committee.

### Protocol

This systematic review was reported according to the Preferred Reporting Items for Systematic Reviews and Meta-Analyses (PRISMA) statement [31]. The study protocol is registered in the International Prospective Register of Systematic Reviews (PROSPERO CRD42021231035). PRISMA Checklist was completed and is available with the Protocol in Additional file 2—Appendix 1.

### Information sources and search strategy

We searched the electronic databases Embase, Global Health Library, Medline and The Cochrane Library for potential studies. The search strategy was built upon prior systematic reviews to identify key search structures and terms such as “informed consent”, “clinical trials” and “electronic informed consent”, and a search string was developed for Embase database consisting of Medical Subject Heading (MeSH) and text words. Search string was adapted to the rest of the databases to account for search syntax, metadata and platform functionality. All search strategies were reviewed by a health science librarian with expertise in systematic review searching. We searched all published and recently completed, and yet to be published studies, and reference lists of relevant systematic reviews from inception to 11 January 2021 in English, Chinese and Spanish. The full search strategy for all databases can be found in Additional file 3—Appendix 2.

### Inclusion criteria

We included studies of all ages and sex that evaluated a consent method within a randomized controlled trial setting (Study Within a Trial – SWAT) [32]. SWATs are defined as “a self-contained research study that has been embedded within a host trial with the aim of evaluating or exploring alternative ways of delivering or organizing a particular trial process” ([32] pp. 1). SWATs are considered the most suitable study design to increase the evidence base for e-IC processes [32].

#### Type of participants

Male and female with no age limit.

#### Type of interventions

One of the three components of IC process (i) information provision, (ii) assessment of participants’ comprehension, (iii) signature must be electronic, whether conducted remotely or face-to-face.

#### Type of comparator

Traditional IC—paper information and consent form.

#### Type of outcome measures

##### Primary outcome

- ◾ Rate of enrolment (defined as the proportion of invited potential participants enrolled and/or the number of participants recruited in a given period (e.g. month).

##### Secondary outcomes

A narrative summary of information on the use of e-IC including:

- ◾ Effects on the economic cost of using e-IC compared to traditional IC
- ◾ Practical benefits and challenges of implementing e-IC, acceptability to potential participants, feasibility, e.g. failure to complete consent process thus needing to switch over to paper consent and other findings on user experience reported on the use of e-IC.

### Exclusion criteria

Book reviews, conference notes, editorials, letters to the editor and abstracts not accompanied by a full text were excluded (Additional file 4 – Appendix 3).

### Study selection

All titles and abstracts were imported to ENDNOTE X9 reference manager and duplicates were removed. The resulting references were uploaded to Covidence Systematic Review manager [33], and further duplicates detected by the system were automatically removed. Titles and abstracts were screened by one reviewer to select studies that fulfilled the eligibility criteria. Full texts were obtained for the short-listed studies and were assessed for final inclusion by two reviewers. For those cases where full text was not available, one attempt was made to contact the authors. Disagreements on whether to include a study were discussed between reviewers and resolved by consensus. Reasons for study exclusion at the full-text stage were recorded and information was summarized using the PRISMA Flow diagram [34]. Additional studies were hand searched by reviewing the reference lists of included studies.

### Risk of bias of included studies

Cochrane risk of bias assessment [35] was performed for each included clinical trial. Risk of bias was assessed on sequence generation, allocation concealment, blinding of participants and personnel, blinding of outcome assessment, incomplete outcome data and selective reporting. Studies were rated as “high risk”, “low risk” or “unclear risk”, and a graphic representation and summary was provided for all included studies. Risk of bias assessment for all Cohort and Case Controlled studies was performed using the Critical Appraisal Skills Programme (CASP) [36] checklist. Risk of bias assessment was performed by one reviewer and verified by a second reviewer. In case of disagreements, consensus was reached by discussion. The potential influence of any risk of bias on the review findings was described.

### Data extraction

Data extraction of selected studies was supported by Covidence Systematic Review Manager, independently performed by one reviewer and verified by a second reviewer. Disagreements were resolved by discussion between the two reviewers. For each included study, data were extracted on the lead author, country in which the study was conducted, study characteristics including design of the SWAT, health topic, health care setting, publication year, sample characteristics including age, sex, inclusion and exclusion criteria, recruitment method into the parent trial—face to face, telephone or online, total number of participants, type of intervention (which component of the IC process was electronic), type of comparator, outcomes, method of outcome assessment and reported findings. Further narrative information was summarized if it was felt to be relevant to the secondary objective of this review. Data extracted were exported to Excel and Review Manager 5.3 for analysing. For studies that had missing data, authors were contacted. A maximum of three email attempts were performed.

### Data analysis and synthesis

A meta-analysis of the quantitative data could not be conducted due to the variety of study designs, comparators and outcome measures. For the primary outcome, quantitative data were summarized descriptively. For the secondary outcomes, as these were not consistently reported by all studies, reviewers categorized study findings and summarized any relevant results descriptively and narratively. SWiM guidelines stated in the protocol could not be used as they are not intended for use in reviews that synthesize qualitative data [37]. Data analysis was performed by one reviewer and verified by the second reviewer using a narrative synthesis approach with thematic summary [38].

### Risk of bias across studies

We planned to assess the overall certainty of evidence of each outcome with the GRADE system, but this could not be done as data could not be pooled. In addition, the main purpose of this systematic review was to summarize the available findings. It was not under the authors’ scope to make recommendations.

## Results

### Study selection

**Fig. 1:**
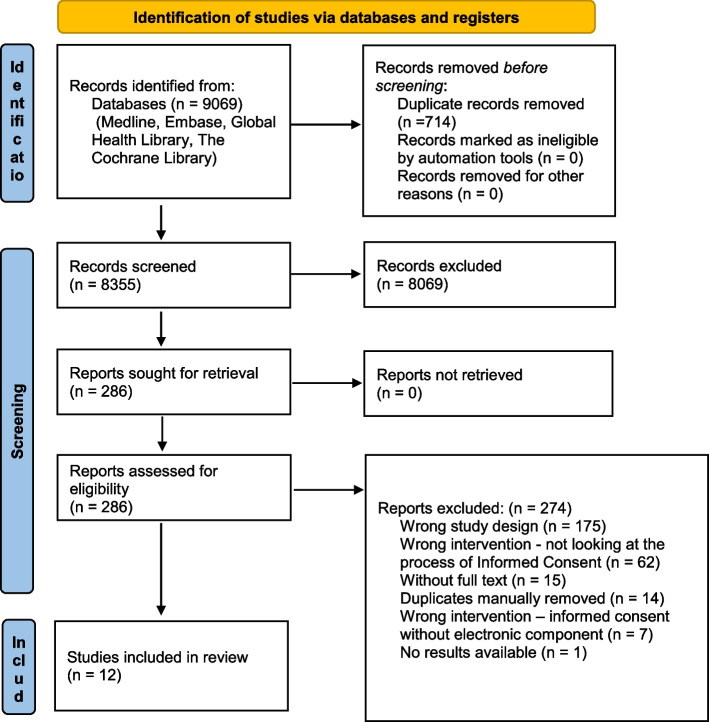
PRISMA flow diagram for this systematic review

The search strategy conducted in all four databases retrieved 9069 records which were imported to Covidence for title and abstract screening. Duplicates were automatically removed by the system. From the resulting 8355 records, a total of 286 references were included in full-text review. Two reviewers assessed the articles independently. Both reviewers planned to assess full text for all studies for final inclusion in the registered protocol. Due to time constraints, the first reviewer assessed 286 articles, and the second reviewer assessed 192 articles (94 remaining articles at full-text stage were assessed only by one reviewer). Twelve studies were included in the final analysis. Study selection and reasons for exclusion are shown in the study flow diagram in Fig. 1. Expanded table of all included studies is shown in Additional file 5 – Appendix 4.

### Study characteristics

A summary of the main characteristics of included studies is shown in Table 1.

**Table 1:** Summary of the main characteristics

Study	Country	Design	Target clinical group	Population	Sample size	Intervention		Comparator	Outcome	Method outcome assessment
						Type	Mode			
Afolabi-2015 [39]	Gambia	Clinical Trial	Malaria	Adults with asymptomatic malaria, < 50 years, more than 50% women with no formal education	311	Video	In person	Paper consent	Comprehension	Comprehension questionnaire administered using laptop computers by trained interviewers who entered participants’ responses to each question
Barrera-2016 [40]	Worldwide	Observational	Pregnant women	Pregnant women, mean age 27.6 years, 81.5% university level education	1179	Online informed consent	In person	None	Comprehension	Four multiple-choice items
Ditai-2018 [41]	Uganda	Observational	Pregnant women	Pregnant women, no mean age reported, 50% with no formal education	30	Slide show using illustrated text on a flip chart or video	In person	Standard researcher read IC	Comprehension and recalling	Modified Quality of Informed Consent form (QuIC) and semi-structured interview
Haussen-2017 [42]	United States	Observational	Stroke	Adults, mean age 73 years	4	DAWN trial: All 3 components of IC electronic. ARISE-II method presumed the same	Remote	Paper consent	Description of first experience with electronic consent	Research Electronic Data Capture
Mattock-2020 [43]	United Kingdom	Clinical Trial	Behavioural problems in young children	Parents mean age 33.59 years, 93% biological mothers, 47% educated to postgraduate level. Mean child age 21.9 months	107	Video as an aid to paper consent	Remote	Paper consent	Recruitment rate	Number of subjects consented in main trial and brief structured interviews
Rothwell-2014 [44]	United States	Clinical Trial	Women, prenatal education	Female participants, 61% had given birth before, 41.94% were educated at a bachelor’s degree	62	Video on iPad as an aid to paper consent	In person	Paper consent	Comprehension	14-item survey and telephone interview
Bobb-2016 [45]	United States	Clinical Trial	Pneumonia	Adults, mean age 55, 49% subjects were male	131	Telemedicine as an aid to paper consent	In person	Paper consent	Comprehension	Quality of informed consent (QuIC) instrument
Dobscha-2005 [46]	United States	Observational	Depression	Adults, 87–93% male. Mean age varied from 57 to 59	31	Videoconferencing—all 3 components electronic	Remote	Paper consent	Description of first experience with electronic consent—Patient satisfaction	12-item mail survey
Dobscha-2005 [46]	United States	Observational	Depression	Adults, 87–93% male. Mean age varied from 57 to 59	31	Videoconferencing—all 3 components electronic	Remote	Paper consent	Description of first experience with electronic consent—Patient satisfaction	12-item mail survey
Jolly-2019 [47]	United Kingdom	Clinical Trial	COPD	Adults, mostly male, mean age 70 years, limited educational qualifications	4214	Standard printed materials with access to a multimedia information resource	Remote	Paper consent	Recruitment rate	Number of patients recruited to host trial and responding invitation
Lurie-2011 [48]	United States	Observational	Spine Surgery	Adults with IDH or SPS—with or without DS. IDH mean age 41.2–42 years, SPS mean age 65.1–67.1 years, IDH female gender 38–46%, SPS female gender 42–52%)	2505	Video decision aid as part of their informed consent process	In person	Before and after viewing video	Changes in treatment preference	Answer to question “What is your current preference for how to treat your spine-related problem?” on a 5-point scale
Swain-2017 [49]	United States	Observational	Breast cancer	Adults with breast cancer, mean age 59 years, 29% attended college or technical school	200	Video	In person	None	Recruitment rate	Patients who signed consent regardless they enrolled in a trial. AIET questionnaire
Weston-1997 [50]	Canada	Clinical Trial	Pregnant women	Pregnant women, median maternal age 31.4–31.8 years, median gestation in weeks 25–27.3, 40–42% college degree or higher	90	Video	In person	Paper consent	Willigness for future participation in a trial	

### Study design and country

From a total of 12 included studies, six were conducted in the USA [42, 44–46, 48, 49], two in the UK [43, 47], one each in Gambia [39], Uganda [41], Canada [50], and one, conducted globally, reporting results from 23 different countries [40].

Study design of all included studies were SWAT, i.e. studies within parent RCTs. Six studies within trials were RCTs [39, 43–45, 47, 50], and six were observational studies [40–42, 46, 48, 49].

### Health topic and setting

Parent studies of included trials addressed different health topics such as depression (2 studies), infectious diseases (3 studies), stroke (1 study), prenatal education (1 study), chronic obstructive pulmonary disease (COPD) (1 study), spine surgery (1 study), breast cancer (1 study), prelabour rupture of membranes (1 study) and prevention of behavioural problems in young children (1 study). Health care settings varied from hospitals, remote community, outpatient clinics, community-based clinics, general practices, physician’s offices and patient’s homes. All studies were published between 2005 and 2020, except one that was published in 1997 [50].

### Study participants

Studies varied in their participants’ characteristics as they targeted selected groups (Table 1). Mean age was reported in 10 studies, ranging from 27 to 73 years [40, 42–50]. Two studies did not provide mean age of participants [39, 41]. Six studies included men and women [39, 42, 45–48]. Four studies included only women [40, 41, 44, 50]. One study did not exclude men in its criteria but represented only female perspective [49] and another study included parents aged ≥ 18 years with child aged between approximately 12 and 36 months [43].

Level of education was reported in nine studies, with three reporting no formal education in the majority of their sample [39, 41, 47]. The remaining six studies reported educational level ranging from university to college education [40, 43, 44, 48–50].

### Recruitment method

Eight of the 12 studies reported their method of recruitment of participation as face-to-face. Other studies reported methods of recruitment were by phone [42, 46], online [40] and by phone and letter [47].

### Sample size

In total, 8864 people participated in the 12 included studies. Number of participants analysed per individual study ranged from 4 to 4214.

### Type of intervention and comparator

#### Intervention type by IC component

Nine out of the 12 included studies only evaluated the first component of the IC process, i.e. electronic information given to trial participants. In these studies, electronic information was provided in different ways, for example, as an aid to the paper IC form [43–45, 47, 48, 50] and as standalone electronic information [39, 41, 49]. Formats for providing information varied from multimedia tool, slide show, video and telemedicine (computer-enabled audio-visual communication). Three studies [40, 42, 46] had all three components of the IC form carried out electronically. No studies evaluated an intervention pertaining to only the second component of IC: participant comprehension. When electronic components of IC were provided, they were done both remotely [42, 43, 46, 47] and on-site/face-to-face [39, 41, 44, 45, 48–50].

#### Comparator

Traditional IC (face-to-face information and paper written consent) was the pre-defined comparator in this systematic review. Eight studies had traditional IC [39, 42–47, 50] as the comparator. Other comparators were used in two studies: standard information read out by researcher [41] and pre- and post-intervention comparison in patient’s preference [48]. Two studies [40, 49] did not include a comparator.

Most studies with a comparator included two arms. One trial [41] included three arms. All comparisons are listed as follows:

#### First component of IC (electronic information giving)

1. Video information vs written versions in local languages or verbal presentation of the written IC given by trained study staff who were native speaker of the local language [39]
2. Slideshow using illustrated text on a flip chart vs video vs standard researcher-read information [41]
3. Video as an aid to paper IC vs written IC [43, 44]
4. Telemedicine (computer-enabled audio-visual communication) as an aid to paper consent vs written IC [45]
5. Written IC with access to a multimedia information resource vs written IC [47]
6. Video decision aid as part of their IC process (before and after comparison in treatment preference) [48]
7. Video vs written IC [50]
8. Educational video vs no comparator [49]

#### All three components of IC were electronic

1. Automated online IC vs no comparator [40]
2. Videoconferencing vs written IC in person [46]
3. Online IC vs written IC [42]

### Study outcomes

Five studies [43, 47, 49] provided data on the primary pre-defined outcome—rate of enrolment. For the secondary pre-defined outcomes, one study described the economic cost of using e-IC but none reported quantitative outcomes on the practical benefits or challenges of implementing e-IC. There was narrative information reported on the acceptability to potential participants. There were outcomes reported by studies that were not anticipated in the protocol: participant’s comprehension of information, effect on changes in treatment preferences by participants, experience of e-IC by users, participant and researcher attitudes towards method of recruitment, number of participants responding to the trial invitation, intention to participate in a clinical trial and retention rates. Outcomes were varied and were measured in different ways. Some studies utilized questionnaires administered through computers, surveys sent by emails, electronic multiple-choice options and in-person or telephone interviews.

Summary of outcomes is shown in Table 2.

**Table 2:** Summary of study outcomes

Study	Sample size *n* = xx Mean age	Educational level	Intervention	Effect on outcome	Other findings
**Primary outcome**					
***Effect on enrolment***					
Bobb 2016 [45]	*n* = 131 Mean age = 55	Not assessed	Telemedicine	No improvement. Computer-enabled audio-visual communication as an aid to paper consent vs written IC: 56% vs. 69%, *p* = 0.142	
Jolly 2019 [47]	*N* = 4214 Mean age = 70	No formal education	Standard printed material with access to multimedia information resource	No improvement. Written IC with access to multimedia resource vs written IC: OR 0.84, 95% CI 0.58 to 1.22	
Mattock 2020 [43]	*N* = 107 Mean parent age = 33.59 Mean child age = 21.9 months	47% educated at postgraduate level	Information video as an aid to patient information sheet	Intervention group less likely to take part in main clinical trial. Video aid to paper v written IC: OR = 0.25, CI = 0.10–0.62, *p* = 0.003	
Swain 2017 [49]	*N* = 200 Mean age = 59	29% attended some college or technical school	Educational video	Improvement on enrolment by 7% post-intervention (13.5% of 200 participants enrolled post-intervention, 6% enrolled pre-intervention, *p* < 0.001)	
Weston 1997 [50]	*N* = 90 Median age = 31.4	40–42% achieved college degree or higher	Information video	Improvement on participants expressing willingness to participate in a future trial (61.9% vs. 35.4%, *χ*^2^ = 6.3; df = 1; *p* = 0.01)	
**Secondary outcomes**					
***Effect on economic costs***					
Afolabi 2015 [39]	*N* = 311 Mean age = NA	> 50% no formal education	Video information	No results available	
Jolly 2019 [47]	*N* = 4214 Mean age = 70	No formal education	Standard printed material with access to multimedia information resource	Additional six people would be recruited per 1000 approached at a cost of £100 per additional patient with the use of an online multimedia intervention. The cost of the online multimedia intervention was estimated £2500	
***Patient comprehension and understanding***					
Afolabi 2015 [39]	*N* = 311 Mean age = NA	> 50% no formal education	Video information	Improvement. Score at day 14: 64%v 40%, *p* = 0.035	
Barrera 2016 [40]	*N* = 1179 Mean age = 27.6	81.5% University level	Online IC	Improvement. Correct understanding of the study’s purpose (86.1%) and correctly identified two of three of the study’s benefits (74.6%). 56% correctly identified some or all of the potential risks of participation	Qualitative interviews in this study supported that the video was easy to understand and improved participants’ attention
Bobb 2016 [45]	*N* = 131 Mean age = 55	Not assessed	Telemedicine	Not inferior to standard face-to-face written consent, measured using a modified quality of informed consent instrument (QuIC) (QuIC scores 74.4 ± 8.1 vs. 74.4 ± 6.9 on a 100-point scale, *p* = 0.999)	
Ditai 2018 [41]	*N* = 30 Mean age = NA	50% no formal education	Slide show using illustrated text on a flip chart	No statistically significant difference on the QuIC tool at 48 h after consenting to any of the three models of IC	Most participants preferred the slide-show message (63%, 19/30), compared with 20% (6/30) for the video message and 17% (5/30) for the standard model
Rothwell 2014 [44]	*N* = 62 Mean age = NA	41.94% bachelor’s degree	Video	Improve understanding of some aspects of a trial: “the alternatives to participation in this study” (4.88 ± 0.42 vs. 4.37 ± 1.10, *p* = .047); “who to contact if you are upset because of participation in this study” (4.41 ± 0.80 vs. 4.03 ± 1.40, *p* = .002); “Whom you should contact if you have questions or concerns about this study” (4.34 ± 0.97 vs. 4.13 ± 1.33, *p* = .009); and “Overall, how well did you understand this study when you signed the consent form” (4.72 ± 0.58 vs. 4.63 ± 0.67, *p* = .019)	Comprehension not inferior to standard face-to-face written consent (QuIC scores 74.4 ± 8.1 vs. 74.4 ± 6.9 on a 100-point scale, *p* = 0.999)
Weston 1997 [50]	*N* = 90 Median age = 31.4	40–42% achieved college degree or higher	Information video	No differences in knowledge about the perinatal trial after receiving a video intervention when compared to written IC but they did find an increase in the retention of knowledge 2–4 weeks later by women in the video intervention group	
***Acceptability to participants***					
Mattock 2020 [43]	*N* = 107 Mean parent age = 33.59 Mean child age = 21.9 months	47% educated at postgraduate level	Information video as an aid to patient information sheet	Positive feedback. Information easy to understand and informative but also commented on additional questions that needed discussing over the phone	Participants in the video group described material as introductory whilst those in standard consent group described the standard information as comprehensive. Participants and researchers found that an initial email contact increased participant’s receptivity to the study and engagement in the trial. Researchers also reported a better understanding of randomization by participants who watched the video
Haussen 2017 [42]	*N* = 4 Mean age = 73	Not assessed	All 3 components electronic for DAWN trial. Method for ARISE-I presumed the same	Acceptability of the use of an entirely electronic IC process to remotely obtain IC from the legally authorized representative (LAR) of stroke patients being enrolled into a clinical trial of neurointervention	
Bobb 2016 [45]	*N* = 131 Mean age = 55	Not assessed	Telemedicine	No significant barriers in the use of telemedicine (computer-enabled audio-visual communication) as an aid to paper consent from its qualitative survey. It reported that video was easy to understand and was better at holding patient’s attention than a paper-based approach would have	
***Changes in treatment preferences***					
Lurie 2011 [48]	*N* = 2505 Mean age = IDH 41.2, SPS 65.1	No difference in education attainment	Video as an aid to the IC	Watching video information prior to enrollment to a clinical trial comparing surgical and non-surgical treatments for spinal diseases led to a shift in treatment preference compared to non-watchers (37.9% vs 20.8%, *p* < 0.0001)	
***Invitation response and retention***					
Jolly 2019 [47]	*N* = 4214 Mean age = 70	No formal education	Standard printed material with access to multimedia information resource	No effect on the proportion of people responding to study invitation (OR = 1.02, 95% CI 0.79 to 1.33) or retention in the trial at 6 (ORs 0.84, 95% CI 0.57 to 1.22) and 12 months after randomization	
Swain 2017 [49]	*N* = 200 Mean age = 59	29% attended some college or technical school	Educational video	Increase by 14% (*p* < .001) in the proportion of patients expressing likelihood to enroll in a trial for breast cancer after the use of an educational video	
***Intervention fidelity***					
Jolly 2019 [47]	*N* = 4214 Mean age = 70	No formal education	Standard printed material with access to multimedia information resource	Number of participants who used the link to access the multimedia resource which was part of the intervention was not reported, so it was unclear how many participants actually used the resource	
Mattock 2020 [43]	*N* = 107 Mean parent age = 33.59 Mean child age = 21.9 months	47% educated at postgraduate level	Information video as an aid to patient information sheet	Utilized an entire remote e-IC process to obtain IC from LAR. However, it was not possible to ascertain whether the LAR actually read the online IC. It was unclear how much time the LARs or patients were given to decide about trial participation	

### Quality assessment

**Fig. 2:**
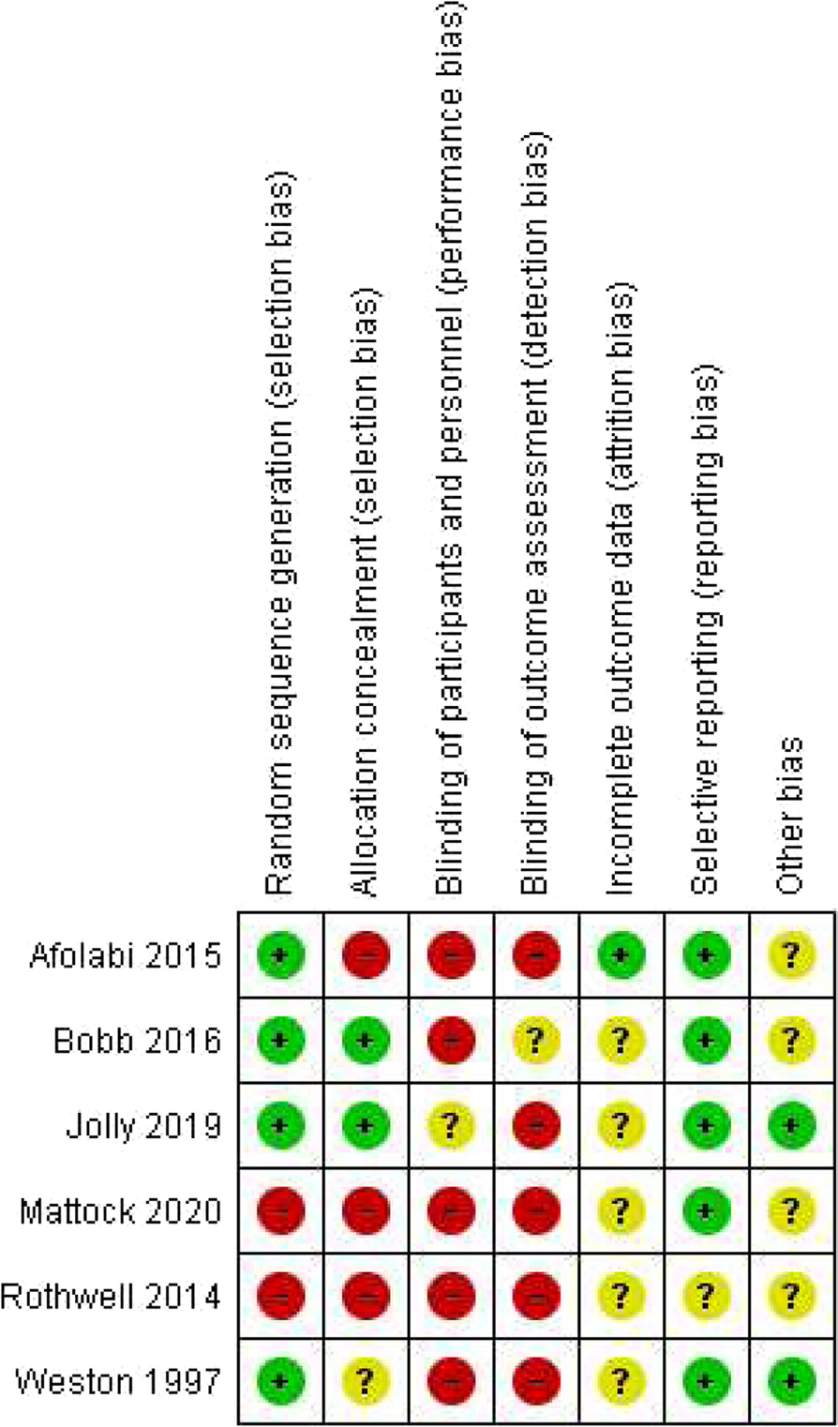
Risk of bias graph: Review authors’ judgements about each risk of bias item presented as percentages across all included studies. Red = high risk, Yellow = unclear risk, Green = low risk

**Fig. 3:**
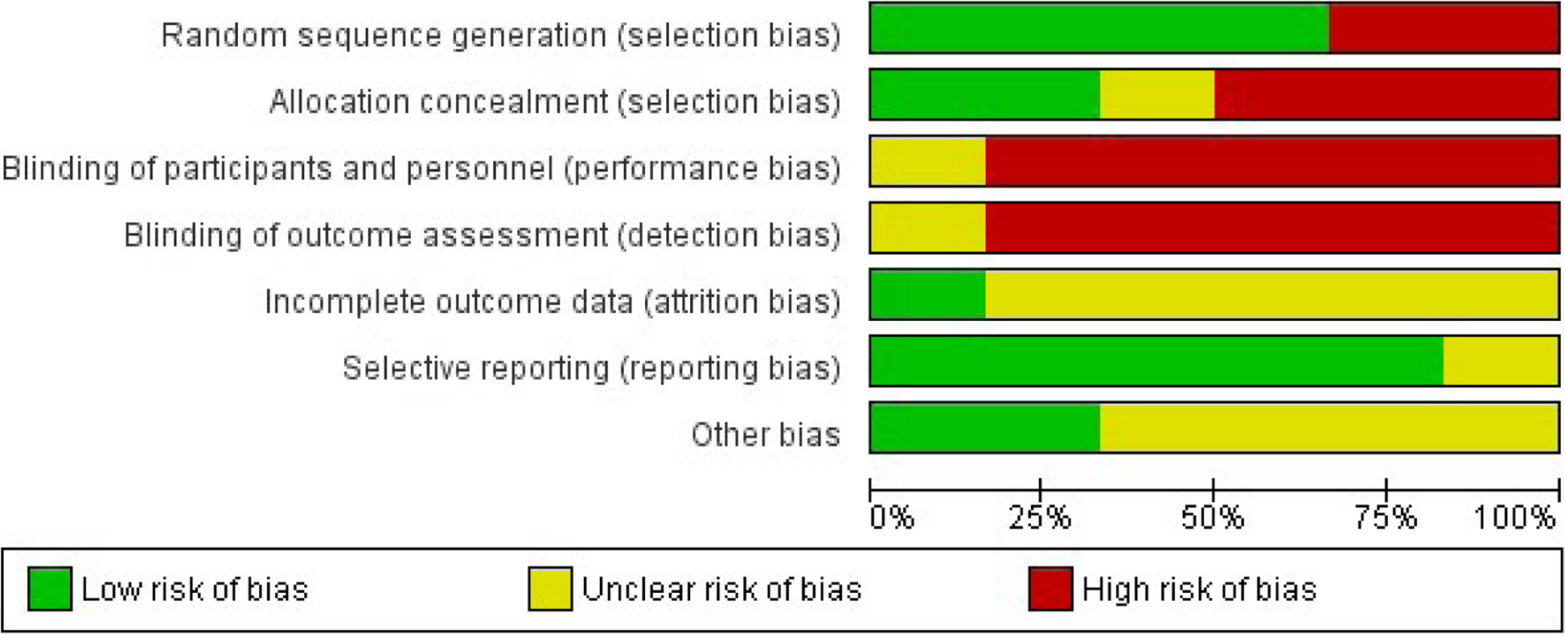
Risk of bias graph: review authors’ judgements about each risk of bias item presented as percentages across all included studies. Red = high risk, Yellow = unclear risk, Green = low risk

The quality of included studies varied. All six RCTs included in this review [39, 43–45, 47, 50] were assessed using the Cochrane Risk of Bias tool, and overall, they were judged to be at moderate-to-high risk of bias. Graphical summary of the risk of bias of included RCTs are shown in Fig. 2 and Fig. 3. Complete assessment of risk of bias using the Cochrane risk of bias table is detailed in Additional files 6 and 9—Appendix 5 and 8.

The assessment of risk of bias for non-randomized studies was performed using the CASP checklist [36]. Templates for the Case Control and Cohort risk of bias are available in Additional files 7 and 8—Appendix 6 and 7. Overall, studies were judged as high risk of bias as there was not sufficient information to perform a detailed assessment. There were a mixture of secondary analyses, case–control studies, cohort studies and studies that lacked objective outcome measures (Additional file 1 and 9—Table S3 and Appendix 8).

### Primary outcome

Five studies provided data on the predefined primary outcome—rate of enrolment, defined as the proportion of invited potential participants enrolled and/or the number of participants recruited in a given period (e.g. month).

#### Effect on recruitment rate

Two studies showed no statistically significant improvement in recruitment rate following an intervention when compared to written IC as controls. These interventions were written IC with access to a multimedia resource [OR 0.84, 95% CI 0.58 to 1.22] [47] and computer-enabled audio-visual communication as an aid to paper consent [56% vs. 69%, *p* = 0.142] [45].

One study showed that an intervention group using a video aid to paper ICF was less likely to take part in the main clinical trial when compared to written IC (OR = 0.25, CI = 0.10–0.62, *p* = 0.003) [43].

Weston et al. showed a significantly larger proportion of participants expressing willingness to participate in a future trial when they have received the video intervention compared to those that received written IC (61.9% vs. 35.4%, *χ*^2^ = 6.3; df = 1; *P* = 0.01) [50]. Swain et al. showed that a video intervention resulted in a statistically significant increase of participant enrolment to a clinical trial by 7% post-intervention when compared to the enrolment rate pre-intervention in a previous year (13.5% of 200 participants enrolled post-intervention, 6% enrolled pre-intervention, *p* < 0.001) [49].

### Secondary outcomes

#### Economic costs

Jolly et al. [47] estimated an additional six people would be recruited per 1000 approached at a cost of £100 per additional patient with the use of an online multimedia intervention which consisted of study-specific information, generic information on, e.g. confidentiality, informed consent, randomization and videos of participants’ experiences. The cost of the online multimedia intervention was estimated £2500 ([47] pp. 4). We contacted the authors for the paper by Afolabi et al. [39], which stated the economic summary of their multimedia intervention was available by correspondence. No reply has been received at the time of writing this report.

### Other secondary outcomes

The predefined secondary outcomes relating to the practical benefits, challenges of implementing e-IC, and acceptability of e-IC to potential participants were not universally reported by all of the studies. Some studies reported findings relevant to these outcomes, so we have provided a descriptive and narrative summary of what we felt were relevant to these outcomes.

#### Patient comprehension and understanding

Five studies [39–41, 44, 45] measured patient’s comprehension and understanding of the information as their primary outcome.

Afolabi et al. [39] reported better comprehension of study information, measured using an IC comprehension questionnaire, at baseline, day 7 and day 14 in the group that received video information when compared to the group that received written versions in local languages or verbal presentation of the written IC by trained native language-speaking staff (score at day 14: 64% vs 40%, *p* = 0.035). Barrera et al. [40] reported that, with the use of an automated IC process for an online trial, a high proportion of participants (*n* = 1,179) showed a correct understanding of the study’s purpose (86.1%) and correctly identified two of three of the study’s benefits (74.6%). Fifty-six percent correctly identified some or all of the potential risks of participation ([40] pp. 5). Rothwell et al. [44] found that using a video presentation on an iPad, followed by a paper copy of the consent form may improve understanding of some aspects of a trial: “the alternatives to participation in this study” (4.88 ± 0.42 vs. 4.37 ± 1.10, *p* = 0.047); “who to contact if you are upset because of participation in this study” (4.41 ± 0.80 vs. 4.03 ± 1.40, *p* = 0.002); “Whom you should contact if you have questions or concerns about this study” (4.34 ± 0.97 vs. 4.13 ± 1.33, *p* = 0.009); and “Overall, how well did you understand this study when you signed the consent form” (4.72 ± 0.58 vs. 4.63 ± 0.67, *p* = 0.019) ([44] pp. 5). Qualitative interviews in this study supported that the video was easy to understand and improved participants’ attention.

Bobb et al. [45] found that comprehension of consent using telemedicine (computer-enabled audio-visual communication as an aid to paper consent) was not inferior to standard face-to-face written consent, measured using a modified quality of informed consent instrument (QuIC) (QuIC scores 74.4 ± 8.1 vs. 74.4 ± 6.9 on a 100-point scale, *p* = 0.999).

Ditai et al. [41] reported no statistically significant difference on the QuIC tool at 48 h after consenting to any of the three models of IC: (i) slideshow using illustrated text on a flip chart, (ii) an approved study video, (iii) standard researcher-read information. Most participants in this study preferred the slideshow message (63%, 19/30), compared with 20% (6/30) for the video message and 17% (5/30) for the standard model.

Weston et al. [50] found no differences in knowledge about the perinatal trial after receiving a video intervention when compared to written IC but they did find an increase in the retention of knowledge 2–4 weeks later by women in the video intervention group.

#### Acceptability to participants and user experiences

Mattock et al. [43] reported positive feedback on the usefulness of a video aid in addition to paper IC in all participants. All 17 participants interviewed found information easy to understand and informative but also commented on additional questions that needed discussing over the phone. Participants in the video group described material as introductory whilst those in standard consent group described the standard information as comprehensive. Participants and researchers found that an initial email contact increased participant’s receptivity to the study and engagement in the trial. Researchers also reported a better understanding of randomization by participants who watched the video.

Haussen et al. [42] reported acceptability of the use of an entirely electronic IC process to remotely obtain IC from the legally authorized representative (LAR) of stroke patients being enrolled into a clinical trial of neurointervention—the DAWN trial ([42] pp.1). The LARs surveyed in this study reported no reservation in using this e-IC process via Research Electronic Data Capture (REDCap) platform, a secure/Health Insurance Portability and Accountability Act-compliant data management platform, developed by the Vanderbilt University. This was used to create an online IC form, which could be accessed on a webpage. The investigator held discussion with the LAR of the potential participant over the telephone. Once agreed to be enrolled, LAR was sent a text message with a link to the webpage to complete the online IC form, which had the capability of capturing the LAR’s electronic signature.

Bobb et al. [45] identified no significant barriers in the use of telemedicine (computer-enabled audio-visual communication) as an aid to paper consent from its qualitative survey. It reported that video was easy to understand and was better at holding patient’s attention than a paper-based approach would have.

### Other outcomes

#### Changes in treatment preferences

Lurie et al. [48] found that watching video information prior to enrolment to a clinical trial comparing surgical and non-surgical treatments for spinal diseases led to a shift in treatment preference compared to non-watchers (37.9% vs 20.8%, *p* < 0.0001). These shifts were balanced and did not demonstrate any overall shift towards or away from surgery.

#### Invitation response and retention rates

Jolly et al. [47] found no effect on the proportion of people responding to study invitation (OR = 1.02, 95% CI 0.79 to 1.33) or retention in the trial at 6 (ORs 0.84, 95% CI 0.57 to 1.22) and 12 months after randomization with the use of a multimedia information resources as an addition to written IC when compared with written IC only (ORs 0.80, 95% CI 0.54 to 1.18).

Study by Swain et al. [49] showed an increase of 14% (*p* < 0.001) in the proportion of patients expressing likelihood to enroll in a trial for breast cancer after the use of an educational video in a survey of attitudes and intention to enroll in therapeutic clinical trials.

#### Assessing intervention fidelity

Jolly et al. [47] did not report the number of participants who used the link to access the multimedia resource which was part of the intervention, so it was unclear how many participants actually used the resource.

Study by Mattock et al. [43] utilized an entirely remote e-IC process to obtain IC from LAR. However, it was not possible to ascertain whether the LAR actually read the online IC. It was unclear how much time the LARs or patients were given to decide about trial participation.

## Discussion

The objective of this systematic review was to investigate the effect of e-IC on enrolment and summarize available research findings on its use. This review has demonstrated that evidence is heterogeneous with varying intervention designs and target populations and disease groups. Narrative synthesis reported inconclusive findings on the impact of the use of electronic consent on enrolment with two of five trials reporting a benefit. We were unable to pool data on the primary outcome as studies had different study designs and comparators and were aimed at different population. Studies were of small sample size, had unclear allocation concealment and had blinding with high risk of bias. The findings from these studies might have limited generalizability as studies that measured the primary outcome were conducted in high-income countries, where access to computers, cell phones and internet is more feasible. Most of the included studies investigated the first component of the IC process, i.e. information given to trial participants. Only three studies evaluated all three components of the IC process.

### Secondary outcomes

#### Patient comprehension and understanding

Many studies reported on participant’s comprehension and recall of information. Though sample sizes, design, population and interventions varied (some interventions were entirely electronic, and others were done as an aid to traditional paper consent, and some of them were administered in person and others remotely), studies described improvement on the use of electronic information on participant’s comprehension and recalling of information. These findings are consistent with findings from another systematic review [25]. Apart from the five studies measuring comprehension and understanding, two additional studies [43, 47] commented on the benefits regarding accurate recalling of study-specific details (what the study was about, benefits and participant’s assignment to different study groups) and a better understanding for all participants in the electronic consent group. Effect on comprehension was of particular interest to studies that included participants from a population that had little or no formal education. These studies showed that for people who were unable to read or write, audio-visual interventions had major positive effects on understanding and recalling. Given the positive findings reported, more studies testing the effect of electronic information without additional aids could be of great interest for the conduct of fully virtual trials.

### Other outcomes

Acceptability of intervention, practical challenges and patient experiences were reported in a variety of ways by these studies, mainly narratively. Promising feedback on e-IC has been obtained, but overall, there was insufficient evidence to enable conclusions to be drawn on patient and/or research staff’s satisfaction on the entire process of e-IC. None of the interventions specifically aimed to assess the second component of the IC process (participant comprehension) and third component of the e-IC process (IC signature). Assessing these specific components could provide further valuable information, especially as there were concerns reported by some participants on the need to interact with research staff to clarify doubts or raise more questions on the information that was provided to them by electronic means.

#### Strengths and limitations of the systematic review and narrative synthesis

This review was a comprehensive and systematic review of the literature, conducted according to the current PRISMA guidelines for the development of its protocol. This protocol of this review was registered in the PROSPERO database. The search strategy was based on prior reviews addressing electronic consent and included broad search terms with no limitation on the year of publication. Authors were contacted for additional information on summary data where applicable, but no responses were received at the time of writing this review.

##### Limitations

Only one author performed title and abstract screening, and due to time constraints, two reviewers independently assessed the full text for only some of the articles, resulting in 94 articles being assessed by only one reviewer. Data extraction and quality assessment was also performed by one reviewer and verified by the second one, but this process was not carried out independently. The CASP checklist for assessing the risk of bias for observational studies in this review was modified by the author to include a scoring method so as to give an idea of the quality of studies. This scoring method has not been validated.

#### Strengths and limitations of included studies

Studies were selected through a robust process following the PRISMA guidelines. All selected trials were embedded within a host trial. Although not all studies had a formal protocol as recommended in the guidelines for SWAT ([32], pp. 2), they all provided valuable data and lessons that could be used for designing future trials to evaluate e-IC, potentially enhancing the processes of conducting more efficient clinical trials.

Some of the included studies were limited by small sample sizes which could potentially lead to chance findings and unreliable conclusions. Not all included SWATs were randomized controlled trials. Some studies were observational, and others were secondary analysis. The lack of comparators or controls increased the risk of bias from confounders. There was a high heterogeneity noted in study design, the target population, type of intervention and comparator among the included studies. While all studies used some form of electronic consent, not all studies tested it remotely. Many trials reported non-objective outcome measures and mainly qualitative data, making it impossible to perform any meta-analysis. The findings of this review were synthesized narratively, which itself carried the risk of bias in reporting due to variation in how researchers summarize narrative findings.

#### Implications and future studies

This review highlights some evidence for improved participant’s understanding and recalling of study information with the use of e-IC.

Information provision and participant understanding are vital components for a valid informed consent. In studies where there is a high risk of potential adverse events associated with an intervention, or if patients have a serious condition, or if the study participants are recruited as inpatients, it is often more practical for investigators and participants to carry out a traditional face-to-face IC process.

In these situations where face-to-face discussion is more practical, e-IC could be used to facilitate the IC process in a number of ways: (i) by presenting accessible study information in digital format, including graphics and multimedia content to aid understanding; (ii) study information can be emailed to participants/ legally appointed representatives for further reading if more time is required to make an informed decision on whether to participate.

The feasibility of IC procedures would vary with different study populations. Electronic processes offer options that may or may not be suitable for the particular study population. It is feasible to replace individual components of the IC process with electronic format, e.g. information provision, electronic informed consent form while retaining some elements of the traditional IC process, e.g. face-to-face discussion prior to signing the electronic consent form.

Hausen et al. [42] demonstrated the feasibility of using an entirely electronic process for all 3 components of IC to recruit acute stroke patients to time-dependent hyperacute stroke treatments.

A few studies have demonstrated the feasibility of conducting all three components of the e-IC remotely in clinical trials. With the development of technologies and the need to conduct clinical trials more efficiently, e-IC could potentially offer a solution to tackle barriers to enrolment, which have been particularly evident during the COVID-19 pandemic.

Different types of e-IC have been developed, described and applied (electronic information given to participants in video, multimedia, assessment of comprehension through questionnaires or surveys, electronic signature, electronic consent face to face, electronic consent through telemedicine) and though many of them show advantages over paper consent with regard to comprehension and recalling, the advantages may be specific to the country where it was tested and its associated socio-economic characteristics, e.g. lack of access to technology such as internet, computers and mobile phones, lower level of literacy. Findings from these studies thus have limited generalizability for global application. Given the heterogeneity of the included studies, this review highlights the need for future high-quality research studies that will evaluate the entire process of e-IC, with detailed description of the three components of the IC process, clearly stated and relevant outcomes such as rate of enrolment, economic benefits, and time taken for e-IC administration. Feasibility of intervention should take into account the characteristics of the target population and the generalizability for the wider population. Qualitative feedback from the investigators and participants could help improve the design for an e-IC process, e.g. user interface, logistical challenges.

Future research of the efficacy of e-IC on recruitment to clinical trials should be built upon robust methodological design, ideally a SWAT that is a clinical trial with suitable comparators to minimize systematic bias. Larger sample sizes are needed to provide sufficient power for precise and reliable conclusions to be drawn on the efficacy of e-IC.

## Conclusion

To our knowledge, this is the first systematic review that considers the definition of electronic consent provided by the FDA and MHRA/HRA guidelines, which is inclusive of all three components of the consent process that are conducted electronically. This review aimed to focus on assessing the relationship between electronic consent and enrolment.

We found few published studies have investigated the impact of e-IC on enrolment and findings were mixed. e-IC may improve participant’s comprehension and recall of information. The heterogeneity of the studies and their high risk of bias meant that it was not possible to provide definitive conclusions on the efficacy of e-IC on enrolment. This review lays the foundation for future research to focus on high-quality studies to evaluate the potential benefit of using e-IC to increase clinical trial enrolment.

## Supplementary Information

**Additional file 1: Table S3.** CASP checklist – Risk of Bias for Cohort and Case control studies. Summarized responses for risk of bias of cohort and case control studies.

**Additional file 2: Appendix 1.** Protocol For A Systematic Review and PRISMA Checklist 2020. Protocol and PRISMA checklist.

**Additional file 3: Appendix 2.** Search strategies by database. **Appendix 2.a.** Embase. **Appendix 2.b.** Medline Ovid. **Appendix 2.c.** Global Health. **Appendix 2.d.** The Cochrane Library.

**Additional file 4: Appendix 3.** Expanded results table of excluded studies at full text and their reasons. All excluded studies at full text with their reasons for exclusion.

**Additional file 5: Appendix 4.** Expanded results table of all included studies. Table containing full results of included studies.

**Additional file 6: Appendix 5.** Cochrane risk of bias table of included RCT studies. Risk of bias with full explanation for included RCT.

**Additional file 7: Appendix 6.** CASP Checklist template for Case Control studies. CASP template used for assessing risk of bias in Case Control studies.

**Additional file 8: Appendix 7.** CASP Checklist template for Cohort studies. CASP template used for assessing risk of bias in Cohort studies.

**Additional file 9: Appendix 8.** Quality Assessment. Complete analysis of Risk of Bias in RCT, Case Control and Cohort studies.

## Data Availability

The datasets used and/or analysed during the current study are available from the corresponding author on reasonable request.

## Abbreviations

COPD: Chronic obstructive pulmonary disease
COVID-19: Coronavirus disease 2019
e-IC: Electronic informed consent
FDA: Food and Drug administration
IC: Informed consent
ICH-GCP: International Conference on Harmonisation-Good Clinical Practice
LAR: Legally authorized representative
MeSH: Medical Subject Heading
QuIC: Modified quality of informed consent instrument
RCT: Randomized controlled trial
SWAT: Studies within a trial
