# Supplementary material for: Electronic informed consent: effects on enrolment, practical and economic benefits, challenges, and drawbacks—a systematic review of studies within randomized controlled trials
Source: Trials. 2023 Feb 21;24:127. doi: 10.1186/s13063-022-06959-6 (PMC9942032; doi:10.1186/s13063-022-06959-6)
Supplement: Supplementary file 1 — Additional file 1: Table S3. CASP checklist – Risk of Bias for Cohort and Case control studies. Summarized responses for risk of bias of cohort and case control studies. [file 13063_2022_6959_MOESM1_ESM.docx]

**Table 3. CASP checklist – Risk of Bias for Cohort and Case control studies**

**Case control studies**

|  | **Barrera-2016** ^50^ | **Haussen-2017** ^39^ | **Dobscha-2005** ^42^ | **Lurie-2011** ^43^ | **Swain-2017**^44^ |
| --- | --- | --- | --- | --- | --- |
| **Section A: Are the results of the trial valid?** | | | | | |
| 1. Did the study address a clearly focused issue? | Yes. | Yes | Yes | Yes | Yes |
| 2. Did the authors use an appropriate method to answer their question? | No. | No | Can´t tell | Can´t tell | Can´t tell |
| **Is it worth continuing?** | | | | | |
| 3. Were the cases recruited in an acceptable way? | Can´t tell | No | Can´t tell | Can´t tell | Can´t tell |
| 4. Were the controls selected in an acceptable way? | No | No | No | No | No |
| 5. Was the exposure accurately measured to minimise bias? | Can´t tell | Yes | Can´t tell | Can´t tell | Can´t tell |
| 6. (a) Aside from the experimental intervention, were the groups treated equally? | No | No | No | No | No |
| 6. (b) Have the authors taken account of the potential confounding factors in the design and/or in their analysis? | No | No | No | No | No |
| **Section B: What are the results?** | | | | | |
| 7. How large was the treatment effect? | Can´t tell | Can´t tell | Can´t tell | Can´t tell | Can´t tell |
| 8. How precise was the estimate of the treatment effect? | Can´t tell | Can´t tell | Can´t tell | Can´t tell | Can´t tell |
| 9. Do you believe the results? | Yes | Can´t tell | Can´t tell | Can´t tell | Can´t tell |
| Section C: Will the results help locally? |  |  |  |  |  |
| 10. Can the results be applied to the local population? | Yes | Yes | Yes | Yes | Yes |
| 11. Do the results of this study fit with other available evidence? | Can´t tell | Can´t tell | Can´t tell | Can´t tell. | Can´t tell |
| **Results** | 3 | 3 | 2 | 2 | 2 |
