# Supplementary material for: Electronic informed consent: effects on enrolment, practical and economic benefits, challenges, and drawbacks—a systematic review of studies within randomized controlled trials
Source: Trials. 2023 Feb 21;24:127. doi: 10.1186/s13063-022-06959-6 (PMC9942032; doi:10.1186/s13063-022-06959-6)
Supplement: Supplementary file 2 — Additional file 2: Appendix 1. Protocol For A Systematic Review and PRISMA Checklist 2020. Protocol and PRISMA checklist. [file 13063_2022_6959_MOESM2_ESM.docx]

Appendix 1: Protocol For A Systematic Review

**ADMINISTRATIVE INFORMATION**

**Title**

Electronic Informed Consent: effects on enrolment, practical and economic benefits, challenges, and drawbacks. Studies within Randomised Controlled Trials.

**Registration**

In accordance with the PRISMA-p reporting guidelines, the systematic review protocol was registered with the International Prospective Register of Systematic Reviews (PROSPERO) on 13 January 2021 and was last updated on XXX (registration number CRD42021231035).

**Authors**

*Contact and affiliations*

Ana Teresita Mazzochi, University of Edinburgh, UK. Email: teremazzochi@gmail.com

Dr Ho-Yan Yvonne Chun, University of Edinburgh, UK. Email: yvonne.chun@ed.ac.uk

Prof Martin Dennis, University of Edinburgh, UK. Email: [martin.dennis@ed.ac.uk](mailto:martin.dennis@ed.ac.uk)

*Corresponding author*

Ana Teresita Mazzochi

Author affiliation: Usher Institute, University of Edinburgh

Physical mailing address: Usher Institute, University of Edinburgh, Old Medical School, Teviot Place, Edinburgh, EH8 9AG

Phone number: +44 (0)131 651 5440

E-mail: teremazzochi@gmail.com

*Contribution of Reviewers*

XXXX is the guarantor, drafted the manuscript and developed the search strategy. HY Chun, M Dennis, and XXXX contributed to the development of the selection criteria, the risk of bias assessment strategy and the data extraction. XXXX screened titles and abstracts and HY Chun acted as second reviewer to solve disagreements on study inclusion. XXXX performed methodological quality assessments, data extraction, data analyses and wrote the initial draft of the manuscript. HY Chun critically reviewed the manuscript. All authors read, provided feedback, and approved the final manuscript.

**Amendment**

In the event a protocol amendment is needed during the conduct of the review, a rationale with the description of the change and date of amendment will be provided.

**Support**

*Internal sources of support*

This study is done as part of the MSc Clinical trials, Usher Institute, University of Edinburgh

*External sources of Support*

None

**INTRODUCTION**

**Rationale**

Enrolment is known to be one of the challenging aspects for achieving successful clinical trials (Frye & Robert, 2020; Gul, 2009; Snowdon et al, 2006). Enrolment is preceded by the process of informed consent (IC), where an effective communication of trial information is crucial for potential participant´s agreement on engaging in a study (Nijhawan et al, 2013).

The notion of IC, along with its essential attributes to consider it valid, i.e., legal competence, voluntariness, information disclosure and comprehension (Nelson-Marten & Rich, 1999), was first introduced in the Nuremberg Code in 1947 (Uncresearch, 2020). The request to obtain a written consent before potential participant´s join a trial, is a requirement of the international guideline for conducting and reporting trials ICH-GCP(R2) (Harmonisation, 2016, pp. 25) and is listed as the first process to carry out in all trials, to ensure that participants are duly informed, and their decision to participate is voluntary and free of undue influence, incentive, or coercion (Mazzochi, 2019).

Ways of carrying out this process have varied in the past 70 years (Wolf et al, 2018), as improved ways to overcome barriers (Taylor, 1999) were sought.

Lockdowns adopted by countries as a response to COVID19 outbreak, impacted trial´s execution by restricting in-person visits. Halt in activities brought on digital health technologies as a viable solution for consenting and recruiting potential participants and as the future approach for clinical research.

Although the trend of resorting to digital technologies as an aid to IC began a few years ago (Gesualdo, 2020), it was not until the global health emergency that these were given serious consideration.

Electronic informed consent (e-IC) is one of the cornerstones for conducting fully remote / virtual trials and though challenges in its implementation were described (Huys et al, 2020), it has also been acknowledged to pose many benefits when compared to paper consent, such as the enhancement in trial recruitment (Chen et al, 2020).

Albeit there is a risk to consider the IC as merely form to be signed to protect participant from harm or institution/investigator from lawsuit (Mazzochi, 2019), IC conceals an underlying complex process (Kadam, 2017) consisting of three components: information provision, participant´s comprehension assessment and signature process (Research, 2020).

Different regulatory authorities issued guidance on the use of e-IC in 2016 (Us_Fda, 2021) and 2018 (Service, 2018) and defined it as “the use of any electronic media (such as text, graphics, audio, video, podcasts or websites) to convey information related to the study and to seek and/or document IC via an electronic device such as a smartphone, tablet or computer” (Agency & Healthcare products, 2018, pp. 4).

IC optimization has been acknowledged by the PRioRiTy trial (Healy et al, 2018) as one of the areas to prioritize when analyzing ways to improve enrolment.

Although e-IC term, can led us think of a process conducted fully electronically, definition in guidelines (Agency & Healthcare products, 2018, pp. 4), considers consent as electronic when any of the 3 components uses electronic means.

While Systematic Reviews on strategies to improve recruitment to randomised controlled trials were conducted, neither of them included e-IC as intervention (Gardner et al, 2020; Treweek et al, 2018b). Other reviews analyzing the impact of digital tools in recruitment are available, though not related to the process of IC (Blatch-Jones et al, 2020; Frampton et al, 2020).

A registered Protocol in PROSPERO for a Study Within a Trial (SWAT), aimed at studying digital presentation of information disclosure, albeit limited the search to one component of the IC process (Duane et al, 2020).

Despite regulatory agencies and varied private-public partnerships (Inc, 2020; Initiative, 2016; Initiative, 2020), acknowledged digital technologies as the future of clinical research, glimpsing its positive effects on recruitment and process quality, e-IC has not yet been globally adopted (Guglielmo, 2020).

**Objectives**

The aim is to investigate the effect of e-IC on enrolment, practical and economic benefits, challenges, and drawbacks of using e-IC through a systematic review. Objectives are:

1. To assess the effects of the use of e-IC on enrolment rate (proportion of invited potential participants enrolled and/or number of participants recruited in a given period (e.g., month)), when used for any of the three components of the consent process: information provision, participant´s comprehension assessment and signature process; compared to traditional IC.
2. To summarize available research findings of the use of e-IC: practical benefits and challenges, acceptability by patients, failure to complete consent process thus needing to switch over to paper consent, or other findings that author may encounter during the review; when used for any of the three components of the consent process; compared to traditional IC.

**METHODS**

**Eligibility Criteria**

*Types of Studies to be included*

Studies of consent method set within a randomized controlled trial (Study Within a Trial – SWAT) (Treweek et al, 2018a).

*Type of Participants*

Male and female with no limit of age.

*Type of Interventions*

Any component of the process of IC (information provision, participant´s comprehension, signature), designed as electronic, whether conducted remotely or face to face

*Type of Comparator*

Traditional IC.

*Type of Outcome measures*

- Primary: rate of enrolment (proportion of invited potential participants enrolled and/or number of participants recruited in a given period (e.g., month))
- Secondary (descriptive):
  - - Effects on economic cost of using e-IC compared to traditional consent
    - Practical benefits and challenges of implementing e-IC, acceptability to potential participants, failure to complete consent process thus needing to switch over to paper consent or other findings reported on the use of e-IC, which will be summarized narratively

No limit will be set for publication date. We will include all English, Chinese and Spanish studies. Book reviews, conference notes, editorials, letters to the editor and abstracts not accompanied by a full text, will be excluded.

**Information Sources**

A search for published and recently completed studies and/or systematic reviews will be conducted across the following electronic databases:

- Embase
- Global Health Library
- Medline – Ovid
- The Cochrane Library

Search strategy will be developed for Embase database using Medical Subject Heading (MeSH) and text words and reviewed by a health science librarian with expertise in systematic reviews searching. Term list will be adapted to the other databases and updated towards the end of the review.

**Search Strategy**

1 "digital informed consent".mp.

2 "digital consent*".mp.

3 "online consent*".mp.

4 "electronic informed consent".mp.

5 "electronic consent*".mp.

6 "dynamic informed consent".mp.

7 "dynamic consent*".mp.

8 "interactive consent*".mp.

9 "econsent*".mp.

10 "e consent*".mp.

11 "online informed consent".mp.

12 "interactive informed consent".mp.

13 1 or 2 or 3 or 4 or 5 or 6 or 7 or 8 or 9 or 10 or 11 or 12

14 exp Multimedia/

15 multimedia.mp.

16 video.mp.

17 exp Telecommunications/

18 telecommunication*.mp.

19 exp "information technology device"/

20 computer*.mp.

21 exp Online Systems/

22 exp Telemedicine/

23 telemedicine.mp.

24 online.mp.

25 electronic.mp.

26 dynamic.mp.

27 digital.mp.

28 interactive.mp.

29 exp informed consent/

30 (consent* or "informed consent*").mp. [mp=title, abstract, heading word, drug trade name, original title, device manufacturer, drug manufacturer, device trade name, keyword, floating subheading word, candidate term word]

31 29 or 30

32 14 or 15 or 16 or 17 or 18 or 19 or 20 or 21 or 22 or 23 or 24 or 25 or 26 or 27 or 28

33 31 and 32

34 13 or 33

35 exp clinical trial/

36 exp randomized controlled trial/

37 "randomized controlled trial*".mp.

38 "controlled clinical trial*".mp.

39 random$.mp.

40 limit 39 to abstracts

41 35 or 36 or 37 or 38 or 40

42 34 and 41

43 limit 42 to (chinese or english or spanish)

**Study records**

*Data Management*

Databases will be searched using pre-defined search strategies, downloading all titles and abstracts to ENDNOTE reference manager. The titles and abstracts will be uploaded to Covidence (Covidence, 2020), a systematic review manager, which will be used for title and abstract screening to select studies that fulfil the eligibility criteria

*Selection process*

Titles and abstracts will be screened by one reviewer against the inclusion criteria. Duplicate studies will be removed. Full text will be obtained for the short-listed studies to be assessed for final inclusion in the review by two reviewers. In the case of missing full text, an attempt will be made to contact authors. Where there is uncertainty on whether to include a study, the two reviewers will discuss and reach a consensus.

Reasons for study exclusion (at the full-text stage) will be recorded and information summarized using the PRISMA Flow diagram (Prisma, 2020).

*Data collection process*

Data extraction and Cochrane risk of bias assessment (Higgins Jpt, 2020) of selected studies will be supported by Covidence.

Data extracted will be independently performed by one reviewer and verified by a second reviewer.

List of included studies with their characteristics, list of excluded studies with their reason, and outcome comparison will be directly exported into Excel and Review Manager 5.3 to summarize results and/or perform a meta-analysis.

Disagreements will be resolved by discussion between two reviewers and in the case of missing information of included studies, authors will be contacted (maximum of three email attempts)*.*

**Data items**

From each included study, we will extract the study title, authors, publication year, type of study, country, healthcare setting, health topic, type of intervention (which component of the process of IC was electronic, how was the process of IC conducted – if face to face or remotely), type of comparator used in the study (description of the process of IC) and outcomes.

**Outcomes and prioritization**

The primary outcome will be the rate of enrolment expressed as a proportion of invited potential participants enrolled and/or the number of participants recruited in a given period (e.g., month).

The secondary outcomes, which we anticipate as descriptive, are:

1. the effects on economic cost of using e-IC when compared to traditional consent (this can include among others: information related to costs of researchers visiting patients, printing costs vs production of electronic information)
2. practical benefits and challenges of implementing e-IC, acceptability to potential participants, failure to complete consent process thus needing to switch over to paper consent or other findings reported on the use of e-IC, which will be summarized narratively

**Risk of bias in individual studies**

Cochrane risk of bias assessment will be performed for each individual included study, based on the information extracted. Areas to analyze will include sequence generation, allocation concealment, blinding of participants and personnel, blinding of outcome assessment, incomplete outcome data and selective reporting. Studies will be rated as “high risk”, “low risk”, “unclear risk” and a graphic representation will be provided for each study.

Judgement will be performed by one reviewer. In case of any doubts, a second reviewer will be consulted, and consensus will be reached by discussion. The potential influence of risk of bias on review findings will be described.

**DATA**

**Synthesis**

If included studies are homogeneous in terms of design and comparators (comparable intervention, population and outcome), a meta-analysis will be conducted for primary outcome rate of enrolment, using a fixed-effects model (Borenstein et al, 2010). I^2^ statistic will be used to assess heterogeneity (0% to 40%: might not be important; 30% to 60%: moderate heterogeneity; 50% to 90%: substantial heterogeneity; 75% to 100%: considerable heterogeneity).

Continuous outcomes will be reported as mean differences and uncertainty expressed as 95% confidence interval (CI), using the Inverse Variance method. If dichotomous outcome data were reported, they will be analyzed as relative risk, using the Mantel-Haenszel method. All results will be expressed with 95%CI and P-value.

If study design and comparators are not homogeneous or heterogeneity is >=50%, a meta-analysis will not be conducted; results for primary and secondary outcomes along with characteristic of studies, will be presented as a narrative synthesis, using the Synthesis Without Meta-Analysis Guidelines (SWiM) (Network, 2020).

**Meta-bias**

Funnel plots will be used to investigate reporting and publication bias when at least ten trials are included in a particular comparison.

**Confidence in cumulative evidence**

The overall quality of the evidence for all outcomes will be assessed based on the GRADE system.

| **PRISMA Checklist 2020** | | | |
| --- | --- | --- | --- |
| **Section and Topic** | **Item #** | **Checklist item** | **Location where item is reported** |
| **TITLE** | | |  |
| Title | 1 | Identify the report as a systematic review. | Page 1 |
| **ABSTRACT** | | |  |
| Abstract | 2 | See the PRISMA 2020 for Abstracts checklist. | Page 5-6 |
| **INTRODUCTION** | | |  |
| Rationale | 3 | Describe the rationale for the review in the context of existing knowledge. | Page 10 |
| Objectives | 4 | Provide an explicit statement of the objective(s) or question(s) the review addresses. | Page 11 |
| **METHODS** | | |  |
| Eligibility criteria | 5 | Specify the inclusion and exclusion criteria for the review and how studies were grouped for the syntheses. | Page 13-14 |
| Information sources | 6 | Specify all databases, registers, websites, organisations, reference lists and other sources searched or consulted to identify studies. Specify the date when each source was last searched or consulted. | Page 12 |
| Search strategy | 7 | Present the full search strategies for all databases, registers and websites, including any filters and limits used. | Appendix 2 |
| Selection process | 8 | Specify the methods used to decide whether a study met the inclusion criteria of the review, including how many reviewers screened each record and each report retrieved, whether they worked independently, and if applicable, details of automation tools used in the process. | Page 13-14 |
| Data collection process | 9 | Specify the methods used to collect data from reports, including how many reviewers collected data from each report, whether they worked independently, any processes for obtaining or confirming data from study investigators, and if applicable, details of automation tools used in the process. | Page 15 |
| Data items | 10a | List and define all outcomes for which data were sought. Specify whether all results that were compatible with each outcome domain in each study were sought (e.g. for all measures, time points, analyses), and if not, the methods used to decide which results to collect. | Page 13-14 |
|  | 10b | List and define all other variables for which data were sought (e.g. participant and intervention characteristics, funding sources). Describe any assumptions made about any missing or unclear information. | Page 13-14 |
| Study risk of bias assessment | 11 | Specify the methods used to assess risk of bias in the included studies, including details of the tool(s) used, how many reviewers assessed each study and whether they worked independently, and if applicable, details of automation tools used in the process. | Page 15 |
| Effect measures | 12 | Specify for each outcome the effect measure(s) (e.g. risk ratio, mean difference) used in the synthesis or presentation of results. | Page 13-14 |
| Synthesis methods | 13a | Describe the processes used to decide which studies were eligible for each synthesis (e.g. tabulating the study intervention characteristics and comparing against the planned groups for each synthesis (item #5)). | Page 15-16 |
|  | 13b | Describe any methods required to prepare the data for presentation or synthesis, such as handling of missing summary statistics, or data conversions. | Page 15-16 |
|  | 13c | Describe any methods used to tabulate or visually display results of individual studies and syntheses. | Page 15-16 |
|  | 13d | Describe any methods used to synthesize results and provide a rationale for the choice(s). If meta-analysis was performed, describe the model(s), method(s) to identify the presence and extent of statistical heterogeneity, and software package(s) used. | Page 15-16 |
|  | 13e | Describe any methods used to explore possible causes of heterogeneity among study results (e.g. subgroup analysis, meta-regression). | Page 15-16 |
|  | 13f | Describe any sensitivity analyses conducted to assess robustness of the synthesized results. | Not Applicable |
| Reporting bias assessment | 14 | Describe any methods used to assess risk of bias due to missing results in a synthesis (arising from reporting biases). | Not Applicable |
| Certainty assessment | 15 | Describe any methods used to assess certainty (or confidence) in the body of evidence for an outcome. | Page 16 |
| **RESULTS** | | |  |
| Study selection | 16a | Describe the results of the search and selection process, from the number of records identified in the search to the number of studies included in the review, ideally using a flow diagram. | Page 17 |
|  | 16b | Cite studies that might appear to meet the inclusion criteria, but which were excluded, and explain why they were excluded. | Appendix 3 |
| Study characteristics | 17 | Cite each included study and present its characteristics. | Page 19-21 |
| Risk of bias in studies | 18 | Present assessments of risk of bias for each included study. | Page 23 |
| Results of individual studies | 19 | For all outcomes, present, for each study: (a) summary statistics for each group (where appropriate) and (b) an effect estimate and its precision (e.g. confidence/credible interval), ideally using structured tables or plots. | Page 26-30 |
| Results of syntheses | 20a | For each synthesis, briefly summarise the characteristics and risk of bias among contributing studies. | Not Applicable |
|  | 20b | Present results of all statistical syntheses conducted. If meta-analysis was done, present for each the summary estimate and its precision (e.g. confidence/credible interval) and measures of statistical heterogeneity. If comparing groups, describe the direction of the effect. | Not Applicable |
|  | 20c | Present results of all investigations of possible causes of heterogeneity among study results. | Not Applicable |
|  | 20d | Present results of all sensitivity analyses conducted to assess the robustness of the synthesized results. | Not Applicable |
| Reporting biases | 21 | Present assessments of risk of bias due to missing results (arising from reporting biases) for each synthesis assessed. | Not Applicable |
| Certainty of evidence | 22 | Present assessments of certainty (or confidence) in the body of evidence for each outcome assessed. | Not Applicable |
| **DISCUSSION** | | |  |
| Discussion | 23a | Provide a general interpretation of the results in the context of other evidence. | Page 31-32 |
|  | 23b | Discuss any limitations of the evidence included in the review. | Page 33 |
|  | 23c | Discuss any limitations of the review processes used. | Page 33 |
|  | 23d | Discuss implications of the results for practice, policy, and future research. | Page 34 |
| **OTHER INFORMATION** | | |  |
| Registration and protocol | 24a | Provide registration information for the review, including register name and registration number, or state that the review was not registered. | Abstract and Page 12 |
|  | 24b | Indicate where the review protocol can be accessed, or state that a protocol was not prepared. | Page 12 |
|  | 24c | Describe and explain any amendments to information provided at registration or in the protocol. | Not Applicable |
| Support | 25 | Describe sources of financial or non-financial support for the review, and the role of the funders or sponsors in the review. | Page 3 |
| Competing interests | 26 | Declare any competing interests of review authors. | Page 3 |
| Availability of data, code and other materials | 27 | Report which of the following are publicly available and where they can be found: template data collection forms; data extracted from included studies; data used for all analyses; analytic code; any other materials used in the review. | Page 3 |
