# Supplementary material for: Electronic informed consent: effects on enrolment, practical and economic benefits, challenges, and drawbacks—a systematic review of studies within randomized controlled trials
Source: Trials. 2023 Feb 21;24:127. doi: 10.1186/s13063-022-06959-6 (PMC9942032; doi:10.1186/s13063-022-06959-6)
Supplement: Supplementary file 3 — Additional file 3: Appendix 2. Search strategies by database. Appendix 2.a. Embase. Appendix 2.b. Medline Ovid. Appendix 2.c. Global Health. Appendix 2.d. The Cochrane Library. [file 13063_2022_6959_MOESM3_ESM.docx]

Appendix 2: Search Strategies

Appendix 2.a: Embase

**Date of export: 11-Jan-2021**

**Timespan: all years**

**Number of results: 5994**

**Search history:**

1 "digital informed consent".mp. 8

2 "digital consent*".mp. 14

3 "online consent*".mp. 80

4 "electronic informed consent".mp. 62

5 "electronic consent*".mp. 102

6 "dynamic informed consent".mp. 9

7 "dynamic consent*".mp. 42

8 "interactive consent*".mp. 2

9 "econsent*".mp. 27

10 "e consent*".mp. 70

11 "online informed consent".mp. 27

12 "interactive informed consent".mp. 8

13 1 or 2 or 3 or 4 or 5 or 6 or 7 or 8 or 9 or 10 or 11 or 12 384

14 exp Multimedia/ 4229

15 multimedia.mp. 8921

16 video.mp. 152057

17 exp Telecommunications/ 78417

18 telecommunication*.mp. 27245

19 exp "information technology device"/ 166035

20 computer*.mp. 1593091

21 exp Online Systems/ 26394

22 exp Telemedicine/ 45626

23 telemedicine.mp. 34651

24 online.mp. 203284

25 electronic.mp. 341814

26 dynamic.mp. 434704

27 digital.mp. 200595

28 interactive.mp. 71759

29 exp informed consent/ 109164

30 (consent* or "informed consent*").mp. [mp=title, abstract, heading word, drug trade name, original title, device manufacturer, drug manufacturer, device trade name, keyword, floating subheading word, candidate term word] 208361

31 29 or 30 208361

32 14 or 15 or 16 or 17 or 18 or 19 or 20 or 21 or 22 or 23 or 24 or 25 or 26 or 27 or 28 2821631

33 31 and 32 26353

34 13 or 33 26378

35 exp clinical trial/ 1552997

36 exp randomized controlled trial/ 637809

37 "randomized controlled trial*".mp. 875315

38 "controlled clinical trial*".mp. 501887

39 random$.mp. 1836929

40 limit 39 to abstracts 1745409

41 35 or 36 or 37 or 38 or 40 2651018

42 34 and 41 6052

43 limit 42 to (chinese or english or spanish) 5994

Appendix 2.b: Medline Ovid

**Date of export: 11-Jan-2021**

**Timespan: all years**

**Number of results: 2154**

**Search history:**

1 "digital informed consent".mp. 4

2 "digital consent*".mp. 4

3 "online consent*".mp. 27

4 "electronic informed consent".mp. 28

5 "electronic consent*".mp. 41

6 "dynamic informed consent".mp. 5

7 "dynamic consent*".mp. 25

8 "interactive consent*".mp. 2

9 "econsent*".mp. 9

10 "e consent*".mp. 36

11 "online informed consent".mp. 9

12 "interactive informed consent".mp. 8

13 1 or 2 or 3 or 4 or 5 or 6 or 7 or 8 or 9 or 10 or 11 or 12 168

14 exp Multimedia/ 1957

15 multimedia.mp. 4934

16 video.mp. 121560

17 exp Telecommunications/ 96377

18 telecommunication*.mp. 6957

19 exp "user computer interface"/ 37698

20 computer*.mp. 782012

21 exp Online Systems/ 15963

22 exp Telemedicine/ 31686

23 telemedicine.mp. 28560

24 online.mp. 96500

25 electronic.mp. 183204

26 dynamic.mp. 281460

27 digital.mp. 111254

28 interactive.mp. 45080

29 exp informed consent/ 41463

30 (consent* or "informed consent*").mp. [mp=title, abstract, original title, name of substance word, subject heading word, floating sub-heading word, keyword heading word, organism supplementary concept word, protocol supplementary concept word, rare disease supplementary concept word, unique identifier, synonyms] 92170

31 29 or 30 92170

32 14 or 15 or 16 or 17 or 18 or 19 or 20 or 21 or 22 or 23 or 24 or 25 or 26 or 27 or 28 1517981

33 31 and 32 9193

34 13 or 33 9208

35 exp clinical trial/ 878188

36 exp randomized controlled trial/ 520643

37 "randomized controlled trial*".mp. 688940

38 "controlled clinical trial*".mp. 121995

39 random$.mp. 1230138

40 limit 39 to abstracts 1165496

41 35 or 36 or 37 or 38 or 40 1566692

42 34 and 41 2187

43 limit 42 to (chinese or english or spanish) 2154

Appendix 2.c: Global Health

**Date of export: 11-Jan-2021**

**Timespan: all years**

**Number of results: 249**

**Search history:**

1 (digital adj8 consent*).mp. 7

2 (online adj8 consent*).mp. 43

3 (electronic adj8 consent*).mp. 21

4 (dynamic adj8 consent*).mp. 4

5 (interactive adj8 consent*).mp. 1

6 "econsent*".mp. 0

7 "e consent*".mp. 3

8 1 or 2 or 3 or 4 or 5 or 6 or 7 78

9 exp multimedia instruction/ 66

10 multimedia.mp. 619

11 video.mp. 4622

12 exp Telecommunications/ 10594

13 telecommunication*.mp. 1387

14 exp "digital technology"/ 411

15 computer*.mp. 20620

16 exp Systems/ 94

17 exp Telemedicine/ 1537

18 telemedicine.mp. 1967

19 online.mp. 18682

20 electronic.mp. 24705

21 dynamic.mp. 21050

22 digital.mp. 7407

23 interactive.mp. 5273

24 exp consent/ 1031

25 (consent* or "informed consent*").mp. [mp=abstract, title, original title, broad terms, heading words, identifiers, cabicodes] 11536

26 24 or 25 11536

27 9 or 10 or 11 or 12 or 13 or 14 or 15 or 16 or 17 or 18 or 19 or 20 or 21 or 22 or 23 102885

28 26 and 27 841

29 8 or 28 843

30 exp clinical trials/ 56910

31 exp randomized controlled trials/ 40012

32 "randomized controlled trial*".mp. 47123

33 "controlled clinical trial*".mp. 3761

34 random$.mp. 195948

35 limit 34 to abstracts 195760

36 30 or 31 or 32 or 33 or 35 207151

37 29 and 36 255

38 limit 37 to (chinese or english or spanish) 249

Appendix 2.d: The Cochrane Library

**Date of export: 11-Jan-2021**

**Timespan: all years**

**Number of results: 2886**

**Search history:**

1 digital informed consent 11

2 digital consent* 3

3 online consent* 47

4 electronic informed consent 28

5 electronic consent* 30

6 dynamic informed consent 0

7 dynamic near/8 consent* 11

8 interactive consent* 2

9 econsent* 31

10 e consent* 162

11 online informed consent 34

12 interactive informed consent 2

13 #1 OR #2 OR #3 OR #4 OR #5 OR #6 OR #7 OR #8 OR #9 OR #10 OR #11 OR 12 306

14 MeSH descriptor: [Multimedia] explode all trees 227

15 (multimedia):ti,ab,kw 1306

16 (video):ti,ab,kw 16152

17 MeSH descriptor: [Telecommunications] explode all trees 6532

18 (telecommunication*):ti,ab,kw 533

19 MeSH descriptor: [Equipment and Supplies] explode all trees 48579

20 (computer):ti,ab,kw 39902

21 MeSH descriptor: [Online Systems] explode all trees 204

22 MeSH descriptor: [Telemedicine] explode all trees 2618

23 (telemedicine):ti,ab,kw 3812

24 (online):ti,ab,kw 14763

25 (electronic):ti,ab,kw 15983

26 (dynamic):ti,ab,kw 13038

27 (digital):ti,ab,kw 11486

28 (interactive):ti,ab,kw 8146

29 MeSH descriptor: [Informed Consent] explode all trees 727

30 (consent OR "informed consent"):ti,ab,kw 82121

31 #29 Or #30 82121

32 #14 OR #15 OR #16 OR #17 OR #18 OR #19 OR #20 OR #21 OR #22 OR #23 OR #24 OR #25 OR #26 OR #27 OR #28 155225

33 #31 AND #32 9806

34 #13 OR #33 9942

35 MeSH descriptor: [Clinical Trials as Topic] explode all trees 48049

36 MeSH descriptor: [Randomized Controlled Trial] explode all trees 119

37 (randomized controlled trial):pt 499052

38 (controlled clinical trial):pt 323982

39 (random$):ab 41873

40 #35 OR #36 OR #37 OR #38 OR #39 614204

41 #34 AND #40 2886
