# Supplementary material for: Electronic informed consent: effects on enrolment, practical and economic benefits, challenges, and drawbacks—a systematic review of studies within randomized controlled trials
Source: Trials. 2023 Feb 21;24:127. doi: 10.1186/s13063-022-06959-6 (PMC9942032; doi:10.1186/s13063-022-06959-6)
Supplement: Supplementary file 4 — Additional file 4: Appendix 3. Expanded results table of excluded studies at full text and their reasons. All excluded studies at full text with their reasons for exclusion. [file 13063_2022_6959_MOESM4_ESM.docx]

Appendix 3: Expanded results table of excluded studies at full text and their reasons

|  | Reference | Rason for exclusion |
| --- | --- | --- |
| 1 | Afolabi-2016-Multimedia tool for obtaining informed consent in the gambia: A mixed method study (Afolabi et al, 2016) | duplicates manually removed |
| 2 | Agre-1994-A randomized trial using videotape to present consent information for colonoscopy (Agre et al, 1994) | wrong study design |
| 3 | Ahluwalia-2014-Enrolling patients in ambulance stroke trials using mobile televideo consent: Preliminary experience (Ahluwalia et al, 2014) | wrong study design |
| 4 | Ahmed-2019-A novel cost-effective patient engagement based educational tool for informed stent insertion consent (Ahmed et al, 2019) | wrong study design |
| 5 | Antommaria-2018-Parents’ Attitudes toward Consent and Data Sharing in Biobanks: A Multi-Site Experimental Survey (Antommaria et al, 2018) | wrong study design |
| 6 | Armijos-2016-MHealth: The use of portable video media versus standard verbal communication in the informed consent (Armijos Leon et al, 2016) | wrong study design |
| 7 | Amstrong-2010-Portable video media for presenting informed consent and wound care instructions for skin biopsies: a randomized controlled trial (Armstrong et al, 2010b) | wrong study design |
| 8 | Amstrong-2010-+/- Informed consent and wound care instructions for skin biopsies: A randomized controlled trial (Armstrong et al, 2010a) | wrong study design |
| 9 | Baenninger-2018-Efficiency of video-presented information about excimer laser treatment on ametropic patients knowledge and satisfaction with the informed consent process (Baenninger et al, 2018) | wrong study design |
| 10 | Bai-2017-Video education on ERCP procedure during informed consent process can improve the awareness of possible risks and complications for patients undergoing ERCP-a prospective, randomized controlled trial (Bai, 2017) | without full text |
| 11 | Bai-2018-Video education can improve awareness of risks for patients undergoing endoscopic retrograde cholangiopancreatography-A randomized trial (Bai & Tian, 2018) | wrong study design |
| 12 | Bai-2019-Video education can improve awareness of risks for patients undergoing endoscopic retrograde cholangiopancreatography-A randomized trial (Bai & Wang, 2019) | wrong study design |
| 13 | Balestra-2016-Social Annotation Valence: The Impact on Online Informed Consent Beliefs and Behavior (Balestra et al, 2016) | wrong study design |
| 14 | Batuyong-2014-Using multimedia to enhance the consent process for bunion correction surgery (Batuyong et al, 2014) | wrong study design |
| 15 | Bell-2006-Can you ask that over the telephone? Conducting sensitive or controversial research using random-digit dialing (Bell et al, 2006) | wrong study design |
| 16 | Bernard-2020-Bringing informed consent to the 21^st^ century – The impact of an online resource and consent process on fertility patient perceptions (Bernard et al, 2020) | wrong study design |
| 17 | Bernard-2020-Emotional Well-Being during Fertility Treatment: A Randomized Controlled Trial to Evaluate the Use of an Online Learning Platform as a Resource (Bernard et al, 2020) | duplicates manually removed |
| 18 | Bethune-2018-e-Consent: approaching surgical consent with mobile technology (Bethune et al, 2018) | wrong study design |
| 19 | Bethune-2018-e-Consent: approaching surgical consent with mobile technology (Bethune et al, 2018) | duplicates manually removed |
| 20 | Bhardwaj-2019-Improving the chemotherapy consent: From paper to tablet (Bhardwaj et al, 2019) | wrong study design |
| 21 | Bickmore-2009-Using computer agents to explain medical documents to patients with low health literacy (Bickmore et al, 2009) | wrong intervention - not looking at the process of IC |
| 22 | Bishop-2002-Client satisfaction in a feasibility study comparing face-to-face interviews with telepsychiatry (Bishop et al, 2002) | wrong intervention - not looking at the process of IC |
| 23 | Blake-2015-Use of mobile devices and the internet for multimedia informed consent delivery and data entry in a pediatric asthma trial: Study design and rationale (Blake et al, 2015) | no results available |
| 24 | Bobb-2016-Telemedicine provides non-inferior research informed consent for remote enrollment: An emergency department-based randomized control trial (Bobb et al, 2016a) | duplicates manually removed |
| 25 | Bollschweiler-2008-Improving informed consent of surgical patients using a multimedia-based program? Results of a prospective randomized multicenter study of patients before cholecystectomy (Bollschweiler et al, 2008) | wrong study design |
| 26 | Book-2020-Access to an online video enhances the consent process, increases knowledge, and decreases anxiety of caregivers with children scheduled for inguinal hernia repair: A randomized controlled study (Book et al, 2020) | wrong study design |
| 27 | Bouhnik-2017-Use of digital technology to boost patient recruitment in inflammatory bowel disease clinical trials (Bouhnik et al, 2017) | wrong intervention - not looking at the process of IC |
| 28 | Bowers-2017-Using a multimedia presentation to improve patient understanding and satisfaction with informed consent for minimally invasive vascular procedures (Bowers et al, 2017) | wrong study design |
| 29 | Bowers-2013-Using a multimedia presentation to improve patient understanding and satisfaction with informed consent for minimally invasive vascular procedures (Bowers et al, 2013) | duplicates manually removed |
| 30 | Brandel-2017-Efficacy of a Procedure-Specific Education Module on Informed Consent in Plastic Surgery (Brandel et al, 2017) | wrong study design |
| 31 | Brubaker-2018-Randomized study of video-enhanced vs. usual research consent process for the E-optimal study (Brubaker et al, 2018) | wrong study design |
| 32 | Brubaker-2019-Recruitment and retention: A randomized controlled trial of video-enhanced versus standard consent processes within the E-OPTIMAL study (Brubaker et al, 2019) | wrong study design |
| 33 | Canagasingham-2018-The role of patient information sheets and portable video media in preventing hospital re-presentations for stent irritation: A single-blinded, randomised controlled trial in a major tertiary hospital (Canagasingham et al, 2018) | wrong study design |
| 34 | Cartmell-2016-Patient participation in cancer clinical trials: A pilot test of lay navigation (Cartmell et al, 2016) | wrong intervention - not looking at the process of IC |
| 35 | Cavazos-2018-Interactive ipad application to enhance the medical disclosure and consent process (Cavazos et al, 2018) | wrong study design |
| 36 | Chalela-2018-Empowering Latina breast cancer patients to make informed decisions about clinical trials: A pilot study (Chalela et al, 2018) | wrong intervention - not looking at the process of IC |
| 37 | Chan-2011-A community-based intervention to promote informed decision making for prostate (Chan et al, 2011) | wrong study design |
| 38 | Chantry-2010-Video versus traditional informed consent for neonatal circumcision (Chantry et al, 2010) | wrong study design |
| 39 | Chapman-2020-Self-directed multimedia process for delivering participant informed consent (Chapman et al, 2020) | wrong study design |
| 40 | Cheung-2014-Consent for blood transfusion: do patients understand the risks and benefits? (Cheung et al, 2014) | wrong study design |
| 41 | Chouinard-2009-Informed consent for videoconsultations in Canada (Chouinard & Scott, 2009) | wrong study design |
| 42 | Chun-2018-Treating anxiety after stroke (task)-protocol for a novel web-enabled randomized controlled trial (Chun et al, 2018) | wrong intervention - not looking at the process of IC |
| 43 | Cornoiu-2011-Multimedia patient education to assist the informed consent process for knee arthroscopy (Cornoiu et al, 2011) | wrong study design |
| 44 | Cowan-2007-Spanish and English Video-Assisted Informed Consent for Intravenous Contrast Administration in the Emergency Department: A Randomized Controlled Trial (Cowan et al, 2007) | wrong study design |
| 45 | Daryl Thornton-2012-Effect of an iPod video intervention on consent to donate organs: a randomized trial (Daryl Thornton et al, 2012) | wrong study design |
| 46 | De Sutter-2020-Implementation of electronic informed consent in biomedical research and stakeholders' perspectives: Systematic review (De Sutter et al, 2020) | wrong study design |
| 47 | Delcambre-2020-Using a Multimedia Tool for Informed Consent in Mohs Surgery: A Randomized Trial Measuring Effects on Patient Anxiety, Knowledge, and Satisfaction (Delcambre et al, 2020) | wrong study design |
| 48 | Deyo-2000-Involving patients in clinical decisions: impact of an interactive video program on use of back surgery (Deyo et al, 2000) | wrong study design |
| 49 | Dharmarajan-2013-Use of a video decision tool to improve informed decision making in hospitalized patients considering palliative radiation therapy (Dharmarajan et al, 2013) | wrong intervention - not looking at the process of IC |
| 50 | Dharmarajan-2019-A Video Decision Aid Improves Informed Decision Making in Patients With Advanced Cancer Considering Palliative Radiation Therapy (Dharmarajan et al, 2019) | wrong intervention - not looking at the process of IC |
| 51 | Ditai-2016-Optimising informed consent for participants in randomised controlled trials: A comparison of three different methods (Ditai et al, 2016) | duplicates manually removed |
| 52 | Donovan-2009-Development of a complex intervention improved randomization and informed consent in a randomized controlled trial (Donovan et al, 2009) | wrong study design |
| 53 | Dunbar-2019-An Educational Video Improves Consent in Pediatric Lumbar Puncture: A Randomized Control Trial (Dunbar et al, 2019) | wrong study design |
| 54 | Dunn-2002-Improving understanding of research consent in middle-aged and elderly patients with psychotic disorders (Dunn et al, 2002) | wrong study design |
| 55 | Dunn-2001-Enhancing comprehension of consent for research in older patients with psychosis: A randomized study of a novel consent procedure (Dunn et al, 2001) | wrong study design |
| 56 | Egekeze-2016-The age of orthoinfo: A randomized controlled trial evaluating patient comprehension of informed consent (Egekeze et al, 2016) | wrong study design |
| 57 | Eilenberg-2004-From informed consent through database lock: An interactive clinical trial conducted using the internet (Eilenberg et al, 2004) | wrong intervention - not looking at the process of IC |
| 58 | El Azem-2014-Evaluation of an interactive multi-media device for delivering information on Le Fort I osteotomy (El Azem et al, 2014) | wrong study design |
| 59 | Ellett-2013-The use of a multimedia module to aid the informed consent process for gynecological laparoscopy for pelvic pain. A randomized control trial (Ellett et al, 2013) | wrong study design |
| 60 | Eyler-2005-A preliminary study of interactive questioning methods to assess and improve understanding of informed consent among patients with schizophrenia (Eyler et al, 2005) | wrong study design |
| 61 | Faisal-2018-Patient Knowledge Improves by Using a Multimedia System for Informed Consent for Inpateint Endoscopy: A Randomized Controlled Trial (Faisal et al, 2018) | wrong study design |
| 62 | Faisal-2018-Patient Knowledge Improves by Using a Multimedia System for Informed Consent for Inpateint Endoscopy: A Randomized Controlled Trial (Faisal et al, 2018) | duplicates manually removed |
| 63 | Faith-2019-Expanding access to research using Tele-health: Lupus and informatics feasibility study (Faith et al, 2019) | wrong study design |
| 64 | Fanaroff-2018-Association of Video-vs. Text-based informed consent with multicenter registry enrollment (Fanaroff et al, 2018a) | wrong study design |
| 65 | Fanaroff-2018-An Observational Study of the Association of Video- Versus Text-Based Informed Consent With Multicenter Trial Enrollment: Lessons From the PALM Study (Patient and Provider Assessment of Lipid Management) (Fanaroff et al, 2018b) | wrong study design |
| 66 | Faura-2019-Informed consent for anaesthesia: Presential or non-presential information? (Faura et al, 2019) | wrong study design |
| 67 | Ferzli-2018-Using a multimedia module to enhance patients' recall of cesarean birth informed consent: A randomized control trial (Ferzli et al, 2018) | wrong study design |
| 68 | Fraval-2015-Internet based patient education improves informed consent for elective orthopaedic surgery: a randomized controlled trial (Fraval et al, 2015) | wrong study design |
| 69 | Friedlander-2009-Improving informed consent in pediatric endoscopy (Friedlander et al, 2009) | wrong study design |
| 70 | Friedlander-2011-A novel method to enhance informed consent: A prospective and randomised trial of form-based versus electronic assisted informed consent in paediatric endoscopy (Friedlander et al, 2011) | wrong study design |
| 71 | Fureman-1997-Evaluation of a video supplement to informed consent: Injection drug users and preventive HIV vaccine efficacy trials (Fureman et al, 1997) | wrong study design |
| 72 | Galve La Hoz-2019-Informed consent (IC), randomized controlled trial digital vs conventional IC (Galve La Hoz et al, 2019) | wrong study design |
| 73 | Gattellari-2005-A community-based randomised controlled trial of three different educational resources for men about prostate cancer screening (Gattellari & Ward, 2005) | wrong study design |
| 74 | Gautschi-2010-Web-based audiovisual patient information system--a study of preoperative patient information in a neurosurgical department (Gautschi et al, 2010) | wrong study design |
| 75 | Gazzinelli-2010-Health education through analogies: preparation of a community for clinical trials of a vaccine against hookworm in an endemic area of Brazil (Gazzinelli et al, 2010) | wrong intervention - not looking at the process of IC |
| 76 | Gitlitz-2017-Employing remote web consenting and social media to facilitate enrollment to an international trial on young lung cancer (Gitlitz et al, 2017) | without full text |
| 77 | Goldberger-2011-Effect of informed consent format on patient anxiety, knowledge, and satisfaction (Goldberger et al, 2011) | wrong study design |
| 78 | Greenberg-2003-How videos can help to inform patients' consent in clinical trials (Greenberg, 2003) | wrong study design |
| 79 | Gunay-2014-Impact of multimedia information on bronchoscopy procedure (Gunay et al, 2014) | wrong study design |
| 80 | Gupta-2018-Audiovisual informed consent process in vaccine trials: Experience from North India (Gupta et al, 2018) | wrong intervention - informed consent without electronic component |
| 81 | Gyomber-2010-Improving informed consent for patients undergoing radical prostatectomy using multimedia techniques: A prospective randomized crossover study (Gyomber et al, 2010) | wrong study design |
| 82 | Hagerott-2019-Lack of parental knowledge of endoscopy procedures after informed consent: Choosing an appropriate learning modality for endoscopy education (Hagerott et al, 2019) | without full text |
| 83 | Hall-2017-Use of Videos Improves Informed Consent Comprehension in Web-Based Surveys Among Internet-Using Men Who Have Sex With Men: A Randomized Controlled Trial (Hall et al, 2017) | wrong study design |
| 84 | Harle-2019-Does an interactive trust-enhanced electronic consent improve patient experiences when asked to share their health records for research? A randomized trial (Harle et al, 2019) | wrong study design |
| 85 | Harmell-2012-Preliminary study of a web-based tool for enhancing the informed consent process in schizophrenia research (Harmell et al, 2012) | wrong study design |
| 86 | Haussen-2020-Legal authorized representative experience with smartphone-based electronic informed consent in an acute stroke trial (Haussen et al, 2020) | duplicates manually removed |
| 87 | Haussen-2019-Legal authorized representative feedback on the use of smartphone platform for electronic informed consent in an acute stroke trial (Haussen et al, 2019) | duplicates manually removed |
| 88 | Hewison-2001-Use of videotapes for viewing at home to inform choice in Down syndrome screening: a randomised controlled trial (Hewison et al, 2001) | wrong intervention - not looking at the process of IC |
| 89 | Hobden-2017-Computer tablet or telephone? A randomised controlled trial exploring two methods of collecting data from drug and alcohol outpatients (Hobden et al, 2017) | wrong intervention - not looking at the process of IC |
| 90 | Hoffner-2012-"Entering a clinical trial: Is it right for you?" - A randomized study of the clinical trials video and its impact on the informed consent process (Hoffner et al, 2012) | wrong study design |
| 91 | Hong-2012-The effect of animation-assisted informed consent using tablet personal computer for contrast-enhanced computed-tomography in emergency department (Hong et al, 2012) | wrong study design |
| 92 | Hopper-1994-Interactive method of informing patients of the risks of intravenous contrast media (Hopper et al, 1994) | without full text |
| 93 | Howard-2019-OSCAA: A feasibility study to inform a randomized controlled trial of an online intervention for young people with skin conditions that affect their appearance (Young Person's Face IT) (Howard et al, 2019) | wrong study design |
| 94 | Huber-2013-Multimedia support for improving preoperative patient education: a randomized controlled trial using the example of radical prostatectomy (Huber et al, 2013) | wrong study design |
| 95 | Isrctn-2017-Effect of FlowOx™ treatment on healing of lower limb ischaemic ulcers (Isrctn, 2017) | wrong intervention - not looking at the process of IC |
| 96 | Isrctn-2019-A very brief face to face intervention, followed by a text message and/or app intervention to support medication adherence in people prescribed treatment for hypertension in primary care (Isrctn, 2019) | wrong intervention - not looking at the process of IC |
| 97 | Jayasinghe-2019-Establishing the Feasibility of a Tablet-Based Consent Process with Older Adults: A Mixed-Methods Study (Jayasinghe et al, 2019) | wrong study design |
| 98 | Jeste-2009-Multimedia consent for research in people with schizophrenia and normal subjects: A randomized controlled trial (Jeste et al, 2009) | wrong study design |
| 99 | Jibaja-2011-Entertainment education for breast cancer surgery decisions: A randomized trial among patients with low health literacy (Jibaja-Weiss et al, 2011) | wrong study design |
| 100 | Johnson-2011-Patient understanding and satisfaction in informed consent for total knee arthroplasty: A randomized study (Johnson et al, 2011) | wrong study design |
| 101 | Joseph-2018-The role of patient information sheets and portable video media in preventing hospital re-presentations for stent irritation: A single-blinded, randomised controlled trial in a major tertiary hospital (Joseph et al, 2018) | duplicates manually removed |
| 102 | Karan-2014-The effect of multimedia interventions on the informed consent process for cataract surgery in rural South India (Karan et al, 2014) | wrong study design |
| 103 | Karunaratne-2010-Improving communication when seeking informed consent: A randomised controlled study of a computer-based method for providing information to prospective clinical trial participants (Karunaratne et al, 2010) | wrong study design |
| 104 | Kinman-2018-Use of an iPadTM application in preoperative counseling for pelvic reconstructive surgery: a randomized trial (Kinman et al, 2018) | wrong study design |
| 105 | Koh-2018-The use of preprocedural educational videos to augment the consenting process and increase patient understanding and satisfaction in interventional radiology (Koh et al, 2018) | without full text |
| 106 | Korfage-2013-Informed choice about breast cancer prevention: randomized controlled trial of an online decision aid intervention (Korfage et al, 2013) | wrong study design |
| 107 | Kraft-2017-A randomized study of multimedia informational aids for research on medical practices: Implications for informed consent (Kraft et al, 2017) | wrong study design |
| 108 | Krishnamurti-2016-A Patient-Centered Approach to Informed Consent: Results from a Survey and Randomized Trial (Krishnamurti & Argo, 2016) | wrong study design |
| 109 | Lairson-2020-Economic evaluation of tailored web versus tailored telephone-based interventions to increase colorectal cancer screening among women (Lairson et al, 2020) | wrong intervention - not looking at the process of IC |
| 110 | Larouche-2015-Mid-you tube slings: A systematic appraisal pf social media on information quality, surgical content and bias about mid-urethral slings (Larouche et al, 2015) | wrong study design |
| 111 | Laskin-2018-Does Viewing a Third Molar Informed Consent Video Decrease Patients' Anxiety? (Laskin et al, 2018) | wrong study design |
| 112 | Lattuca-2018-Impact of video on the understanding and satisfaction of patients receiving informed consent before elective inpatient coronary angiography: a randomized trial (Lattuca et al, 2018) | wrong study design |
| 113 | Le Blanc-2018-Patient satisfaction with the consent discussion is not improved by showing patients their computed tomography or angiography images before they undergo vascular surgery (LeBlanc et al, 2018) | wrong study design |
| 114 | Leclercq-2010-A review of surgical informed consent: past, present, and future. A quest to help patients make better decisions (Leclercq et al, 2010) | wrong study design |
| 115 | Leon-2017-Improvement of informed consent in urological surgeries: Portable video media versus standard verbal communication. A randomized controlled trial (Leon et al, 2017) | without full text |
| 116 | Li-2020-Effect of video-assisted education on informed consent and patient education for peripherally inserted central catheters: a randomized controlled trial (Li et al, 2020) | wrong study design |
| 117 | Lin-2018-Educational video-assisted versus conventional informed consent for trauma-related debridement surgery: a parallel group randomized controlled trial(Lin et al, 2018) | wrong study design |
| 118 | Lindsley-2019-Improving quality of the informed consent process: Developing an easy-to-read, multimodal, patient-centered format in a real-world setting (Lindsley, 2019) | wrong study design |
| 119 | Love-2015-Video-based education for basal cell carcinoma treatment: A pilot study (Love et al, 2015) | duplicates manually removed |
| 120 | Love-2016-A video-based educational pilot for basal cell carcinoma (BCC) treatment: A randomized controlled trial (Love et al, 2016) | wrong intervention - not looking at the process of IC |
| 121 | Mack-2018-The use of a counselling video during an oral immunotherapy counselling session to improve parent and patient knowledge (Mack & Hanna, 2018) | wrong study design |
| 122 | Madeira-2018-Using the EngagedMD multimedia platform to improve informed consent for ovulation induction, intrauterine insemination, and in vitro fertilization (Madeira et al, 2018) | wrong study design |
| 123 | Man-2015-Improving recruitment to a study of telehealth management for long-term conditions in primary care: two embedded, randomised controlled trials of optimised patient information materials (Man et al, 2015) | wrong intervention - informed consent without electronic component |
| 124 | Manne-2010-Facilitating informed decisions regarding microsatellite instability testing among high-risk individuals diagnosed with colorectal cancer (Manne et al, 2010) | wrong study design |
| 125 | Mapstone-2007-Strategies to improve recruitment to research studies (Mapstone et al, 2007) | wrong study design |
| 126 | Mason-2003-The use of video information in obtaining consent for female sterilisation: A randomised study (Mason et al, 2003) | wrong study design |
| 127 | Mawhinney-2019-Oxford Video Informed Consent Tool (OxVIC): a pilot study of informed video consent in spinal surgery and preoperative patient satisfaction (Mawhinney et al, 2019) | wrong study design |
| 128 | Mayhew-2020-Interactive group-based orientation sessions: A method to improve adherence and retention in pragmatic clinical trials (Mayhew et al, 2020) | wrong intervention - not looking at the process of IC |
| 129 | Mayilvaganan-2018-Comparison of the efficacy of three different methods of explaining the surgical procedure of hemithyroidectomy (Mayilvaganan & Shivaprasad, 2018) | wrong study design |
| 130 | McCormack-2019-Supporting informed clinical trial decisions: Results from a randomized controlled trial evaluating a digital decision support tool for those with intellectual disability (McCormack et al, 2019) | wrong study design |
| 131 | McDonald-2011-Single-center multi-study icu research using a computerized clinical information system (McDonald et al, 2011) | without full text |
| 132 | McEvoy-2015-An interactive video improves the understanding of a hypothetical adaptive acute stroke trial (McEvoy et al, 2015) | without full text |
| 133 | McMorris-2004-A comparison of methods to obtain active parental consent for an international student survey (McMorris et al, 2004) | wrong intervention - informed consent without electronic component |
| 134 | McTiernan-1995-Informed consent in the Women's Health Initiative clinical trial and observational study (McTiernan et al, 1995) | wrong intervention - not looking at the process of IC |
| 135 | Moore-2021-Does the use of video improve patient satisfaction in the consent process for local-anaesthetic urological procedures? (Moore et al, 2021) | wrong study design |
| 136 | Moseley-2006-Effects of presentation method on the understanding of informed consent (Moseley et al, 2006) | wrong study design |
| 137 | Naef-2014-Ethics procedures and patient informed consent in a European-wide multi-centre study (Naef et al, 2014) | wrong intervention - not looking at the process of IC |
| 138 | Nalavenkata-2016-The use of portable video media versus standard verbal communication in the urological consent process: A randomised controlled clinical trial (Nalavenkata et al, 2016) | duplicates manually removed |
| 139 | Naqvi-2014-A prospective, randomized trial of informed consent for cardiopulmonary resuscitation (Naqvi et al, 2014) | wrong intervention - not looking at the process of IC |
| 140 | Nct-2019-Use of Videos to Improve Patient Knowledge on Prenatal Genetics (Nct, 2019b) | wrong study design |
| 141 | Nct-2019-Trial to Compare eConsent With Standard Consent Among Prospective Biobank Participants (Nct, 2019a) | wrong study design |
| 142 | Nct-2020-Use of Consent Language and Mode to Improve Interactive Voice Response Survey in Colombia and Uganda (Nct, 2020) | wrong intervention - not looking at the process of IC |
| 143 | Neary-2010-The benefits of an interactive, individualized online patient pathway for patients undergoing minimally invasive radioguided parathyroidectomy: A prospective, double-blinded, randomized clinical trial (Neary et al, 2010) | wrong intervention - not looking at the process of IC |
| 144 | Nehme-2013-The use of multimedia consent programs for surgical procedures: A systematic review (Nehme et al, 2013) | wrong study design |
| 145 | Neuner-2015-Randomized controlled trial of a decision aid with tailored fracture risk tool delivered via a patient portal (Neuner et al, 2015) | wrong intervention - not looking at the process of IC |
| 146 | Nishimura-2013-Improving understanding in the research informed consent process: a systematic review of 54 interventions tested in randomized control trials (Nishimura et al, 2013) | wrong study design |
| 147 | Nodora-2016-High rates of informed consent for biospecimen and data sharing among low-income Hispanic women: Results of a pilot randomized trial (Nodora et al, 2016a) | wrong intervention - not looking at the process of IC |
| 148 | Nodora-2016-High rates of informed consent for biospecimen sharing among hispanic women in a safety-net clinic (Nodora et al, 2016b) | wrong study design |
| 149 | Noroozi-2017-Improving stroke clinical trial enrollment via the use of telemedicine (Noroozi & Sangha, 2017) | wrong study design |
| 150 | Nwaru-2016-Building a recruitment database for asthma trials: A conceptual framework for the creation of the UK Database of Asthma Research Volunteers (Nwaru et al, 2016) | wrong intervention - not looking at the process of IC |
| 151 | Odackal-2020-Video-assisted consent in a randomized clinical trial: Impact on subject recruitment, knowledge, and satisfaction (Odackal et al, 2020) | without full text |
| 152 | Offerman-2013-The use of delayed telephone informed consent for observational emergency medicine research is ethical and effective (Offerman et al, 2013) | wrong study design |
| 153 | O´Lonergan-2011-Novel approach to parental permission and child assent for research: Improving comprehension (O'Lonergan & Forster-Harwood, 2011) | wrong study design |
| 154 | Olsen-2017-Patient Recall of Information on a Third Molar Informed Consent Video (Olsen et al, 2017) | wrong study design |
| 155 | Olver-2009-Improving informed consent to chemotherapy: A randomized controlled trial of written information versus an interactive multimedia CD-ROM (Olver et al, 2009) | wrong study design |
| 156 | Omezli-2020-Does Watching Videos Increase the Perioperative Anxiety in Patients Undergoing Third Molar Surgery? A Randomized Trial (Omezli et al, 2020) | wrong study design |
| 157 | Padival-2019-Addition of a Video to Informed Consent for Colonoscopy Improves Patient Retention and Satisfaction: Results of a Randomized Controlled Trial (Padival et al, 2019) | wrong study design |
| 158 | Pallett-2016-Informed consent for hysterectomy: Does a video presentation improve patient comprehension? (Pallett et al, 2016) | wrong study design |
| 159 | Pallett-2018-A randomized controlled trial to determine whether a video presentation improves informed consent for hysterectomy (Pallett et al, 2018) | wrong study design |
| 160 | Palmer-2018-Multimedia Aided Consent for Alzheimer's Disease Research | wrong study design |
| 161 | Park-2015-Prospective randomized controlled study on the efficacy of multimedia informed consent for patients who are scheduled to receive greenlight HPS photoselective vaporization of the prostate surgery (Park et al, 2015) | wrong study design |
| 162 | Parke-2016-Health in our hands: Using tablet technology to connect patients to research (Parke et al, 2016) | wrong intervention - not looking at the process of IC |
| 163 | Patel-2009-Improving informed consent of surgical patients using a multimedia-based program?: Results of a prospective randomized multicenter study of patients before cholecystectomy (Patel et al, 2009) | wrong study design |
| 164 | Pawlak-2015-Orthodontic informed consent considering information load and serial position effect (Pawlak et al, 2015) | wrong study design |
| 165 | Pekmezaris-2020-Adapting a home telemonitoring intervention for underserved Hispanic/Latino patients with type 2 diabetes: an acceptability and feasibility study (Pekmezaris et al, 2020) | wrong intervention - not looking at the process of IC |
| 166 | Penn-2009-Informed consent and aphasia: Evidence of pitfalls in the process (Penn et al, 2009) | wrong study design |
| 167 | Pepper-2017-Use of pre-anesthetic informed consent video to facilitate understanding and anxiolysis in patients undergoing regional anesthesia for surgical procedures (Pepper et al, 2017) | wrong study design |
| 168 | Peralta-2017-A pilot pragmatic randomized trial of CKD screening to improve care among hypertensive veterans (Peralta et al, 2017) | wrong intervention - not looking at the process of IC |
| 169 | Perdue-2011-Data collection and storage for online screening prior to consent (Perdue et al, 2011) | wrong intervention - not looking at the process of IC |
| 170 | Pereira-2021-Rationale and design of the TAILOR-PCI digital study: Transitioning a randomized controlled trial to a digital registry (Pereira et al, 2021) | wrong intervention - not looking at the process of IC |
| 171 | Perez-2019-Large-scale assessment of a smartwatch to identify atrial fibrillation (Perez et al, 2019) | wrong intervention - not looking at the process of IC |
| 172 | Perkins-1998-An examination of self- and telephone-administered modes of administration for the Australian SF-36 (Perkins & Sanson-Fisher, 1998) | wrong intervention - not looking at the process of IC |
| 173 | Perni-2019-Assessment of Use, Specificity, and Readability of Written Clinical Informed Consent Forms for Patients with Cancer Undergoing Radiotherapy (Perni et al, 2019) | wrong intervention - not looking at the process of IC |
| 174 | Perrault-2018-Seeking Ways to Inform the Uninformed: Improving the Informed Consent Process in Online Social Science Research (Perrault & Keating, 2018) | wrong intervention - not looking at the process of IC |
| 175 | Perrenoud-2015-The effectiveness of health literacy interventions on the informed consent process of health care users: A systematic review protocol (Perrenoud et al, 2015) | wrong study design |
| 176 | Phillippe-2006-Effects of video information in patients undergoing coronary angiography (Philippe et al, 2006) | wrong study design |
| 177 | Piano-2010-Stroke awareness among stroke victors: A randomized open label clinical trial comparing video based stroke education program vs. nurse education (Piano et al, 2010) | wrong intervention - not looking at the process of IC |
| 178 | Plante-2018-Recruitment of seniors to a randomized con-trolled trial using electronic health record-enabled patient portal recruitment messages (Plante et al, 2018) | wrong intervention - not looking at the process of IC |
| 179 | Pletcher-2014-Informed consent in randomized quality improvement trials: A critical barrier for learning health systems (Pletcher et al, 2014) | wrong study design |
| 180 | Porter-2020-Enhancing the Informed Consent Process Using Shared Decision Making and Consent Refusal Data from the CLEAR III Trial (Porter et al, 2020) | wrong intervention - informed consent without electronic component |
| 181 | Prescott-1999-Factors that limit the quality, number and progress of randomised controlled trials (Prescott et al, 1999) | wrong study design |
| 182 | Price-2019-SMOOTH: Self-Management of Open Online Trials in Health analysis found improvements were needed for reporting methods of internet-based trials (Price et al, 2019) | wrong intervention - not looking at the process of IC |
| 183 | Prochazka-2014-Patient perceptions of surgical informed consent: is repeat back helpful or harmful? (Prochazka et al, 2014) | wrong intervention - informed consent without electronic component |
| 184 | Qi-2018-Construction of integrated model of thrombolysis for acute ischemic stroke (Qi et al, 2018) | wrong intervention - not looking at the process of IC |
| 185 | Quevedo-2018-Informed consent comprehension among vulnerable populations in Ecuador: video-delivered vs. in-person standard method (Quevedo et al, 2018) | wrong study design |
| 186 | Rafaloff-2019-Understanding trial specific recruitment challenges - A dynamic approach to identifying and overcoming obstacles: PD patient's perspective (Rafaloff et al, 2019) | wrong intervention - not looking at the process of IC |
| 187 | Rahimpour-2019-Utility of a mobile clinical decision support application in patients undergoing spinal cord stimulation (Rahimpour et al, 2019) | wrong intervention - not looking at the process of IC |
| 188 | Rai-2020-A rationale and framework for seeking remote electronic or phone consent approval in endovascular stroke trials-special relevance in the COVID-19 environment and beyond (Rai & Frei, 2020) | wrong study design |
| 189 | Rana-2020-A randomized controlled trial of video-education or in-person genetic counseling for men with prostate cancer (ProGen) (Rana et al, 2020) | wrong study design |
| 190 | Rangel-2002-Development of an internet-based protocol to facilitate randomized clinical trials in pediatric surgery (Rangel et al, 2002) | wrong study design |
| 191 | Ranta-2014-TIA and minor stroke utility of primary care based tia electronic decision support: A cluster randomized controlled trial (Ranta et al, 2014) | wrong intervention - not looking at the process of IC |
| 192 | Ratan-2018-Multimedia versus traditional informed consent for cesarean delivery: A randomized controlled trial (Ratan et al, 2018) | without full text |
| 193 | Reder-2017-Does a decision aid improve informed choice in mammography screening? Results from a randomised controlled trial (Reder & Kolip, 2017) | wrong study design |
| 194 | Relton-2019-Health system trials (Relton et al, 2019) | wrong study design |
| 195 | Richardson-2018-A video depicting resuscitation did not impact upon patients' decision-making (Richardson-Royer et al, 2018) | wrong study design |
| 196 | Richardson-2015-A prospective, randomized trial of informed consent for cardiopulmonary resuscitation-informing with or without video (Richardson-Royer et al, 2015) | wrong study design |
| 197 | Riga-2019-Support through Mobile Messaging and digital health Technology for Diabetes (SuMMiT-D) feasibility trial: Strategies and data collection for monitoring of rapid recruitment in a primary care setting (Riga et al, 2019) | wrong intervention - not looking at the process of IC |
| 198 | Rioja-2016-Informed consent: Conventional informed consent versus digital informed consent: Results of a randomized controlled trial (in Spain) (Rioja et al, 2016) | without full text |
| 199 | Roberts-2016-Testing with feedback improves recall of information in informed consent: A proof of concept study (Roberts et al, 2016) | wrong study design |
| 200 | Robinson-2015-Increasing participation in research-breast cancer (Robinson & Swain, 2015) | duplicates manually removed |
| 201 | Robinson-2016-Increasing participation in research-breast cancer (Robinson & Swain, 2016) | without full text |
| 202 | Rocheleau-2010-Effect of survey instrument on participation: A randomization study ofa mailed questionnaire versus a computer-assisted telephone interview (Rocheleau et al, 2010) | wrong intervention - not looking at the process of IC |
| 203 | Rogers-2019-Does the provision of a DVD-based audio-visual presentation improve recruitment in a clinical trial? A randomised trial of DVD trial invitations (Rogers et al, 2019) | wrong intervention - not looking at the process of IC |
| 204 | Roos-2002-In vivo dental plaque pH variation with regular and diet soft drinks (Roos & Donly, 2002) | wrong intervention - not looking at the process of IC |
| 205 | Rosenbaum-2011-An internet-based, multimedia informed consent resource for proton therapy clinical trials: A pilot study (Rosenbaum et al, 2011) | without full text |
| 206 | Rosenheck-2011-Challenges in the design and conduct of controlled clinical effectiveness trials in schizophrenia (Rosenheck et al, 2011) | wrong intervention - not looking at the process of IC |
| 207 | Rossi-2004-Informed consent for ankle fracture surgery: Patient comprehension of verbal and videotaped information (Rossi et al, 2004) | wrong study design |
| 208 | Rossi-2005-Video informed consent improves knee arthroscopy patient comprehension (Rossi et al, 2005) | wrong study design |
| 209 | Rostom-2002-A randomized trial of a computerized versus an audio-booklet decision aid for women considering post-menopausal hormone replacement therapy (Rostom et al, 2002) | wrong intervention - not looking at the process of IC |
| 210 | Rothwell-2020-Comparison of Video, App, and Standard Consent Processes on Decision-Making for Biospecimen Research: A Randomized Controlled Trial (Rothwell et al, 2020) | wrong study design |
| 211 | Rowbotham-2013-Interactive Informed Consent: Randomized Comparison with Paper Consents (Rowbotham et al, 2013) | wrong study design |
| 212 | Rowlands-2017-Recruitment to randomised controlled trials in patients receiving unplanned hospital care: A systematic review and in-depth analysis using the ORRCA database (Rowlands et al, 2017) | wrong study design |
| 213 | Rustveld-2011-A patchwork of life: A bilingual breast cancer treatment patient decision aid targeted at medically underserved women (Rustveld & Jibaja-Weiss, 2011) | wrong intervention - not looking at the process of IC |
| 214 | Ruth-2020-An Electronic Data Capture Framework (ConnEDCt) for global and public health research: Design and implementation (Ruth et al, 2020) | wrong intervention - not looking at the process of IC |
| 215 | Rutkove-2017-Displaced reality: The challenges of creating an ALS clinical study in which all data collection takes place in the patient's home (Rutkove & Shefner, 2017) | wrong study design |
| 216 | Ryan-2008-Audio-visual presentation of information for informed consent for participation in clinical trials(Ryan et al, 2008) | wrong study design |
| 217 | Saglam-2020-Educational Video Addition to the Bariatric Surgery Informed Consent Process: a Randomized Controlled Trial (Saglam et al, 2020) | wrong study design |
| 218 | Sahai-2006-Video consent: a pilot study of informed consent in laparoscopic urology and its impact on patient satisfaction (Sahai et al, 2006) | wrong study design |
| 219 | Sanderson-2017-Public Attitudes toward Consent and Data Sharing in Biobank Research: A Large Multi-site Experimental Survey in the US (Sanderson et al, 2017) | wrong intervention - not looking at the process of IC |
| 220 | Sano-2011-Alzheimer disease cooperative study (ADCS) home based assessment: Designing trials with new technology (Sano et al, 2011) | wrong intervention - not looking at the process of IC |
| 221 | Sanossian-2009-Simultaneous ring voice-over-Internet phone system enables rapid physician elicitation of explicit informed consent in prehospital stroke treatment trials (Sanossian et al, 2009) | wrong study design |
| 222 | Sariturk-2017-Effectiveness of Visual Methods in Information Procedures for Stem Cell Recipients and Donors (Sarıtürk et al, 2017) | wrong study design |
| 223 | Saver-2014-Methodology of the Field Administration of Stroke Therapy - Magnesium (FAST-MAG) phase 3 trial: Part 2 - prehospital study methods (Saver et al, 2014) | wrong intervention - informed consent without electronic component |
| 224 | Schauer-2019-Video or verbal? A randomised trial of the informed consent process prior to endoscopy (Schauer et al, 2019) | wrong study design |
| 225 | Schenker-2011-Interventions to improve patient comprehension in informed consent for medical and surgical procedures: a systematic review (Schenker et al, 2011) | wrong study design |
| 226 | Schlechtweg-2014-iPad-based patient briefing for radiological examinations-a clinical trial (Schlechtweg et al, 2014) | wrong intervention - not looking at the process of IC |
| 227 | Schneider-2020-Design of a virtual longitudinal observational study in Parkinson's disease (AT-HOME PD) (Schneider et al, 2020) | wrong intervention - not looking at the process of IC |
| 228 | Valenza-2014-Assessing the benefit of a personalized EHR-generated informed consent in a dental school setting (Valenza et al, 2014) | wrong study design |
| 229 | Schover-2019-Pragmatic trial of an online self-help intervention for sexual problems after cancer (Schover et al, 2019) | wrong intervention - not looking at the process of IC |
| 230 | Schwartze-2013-Design and implementation of an informed consent process for a standardized health information exchange solution on the example of the lower saxony bank of health (Schwartze et al, 2013) | wrong study design |
| 231 | Sharkir-2020-What information do patients recall from the third molar surgical consultation? (Shakir et al, 2020) | wrong study design |
| 232 | Shelton-2015-A computer-based education intervention to enhance surrogates' informed consent for genomics research (Shelton et al, 2015) | wrong study design |
| 233 | Shimomura-2017-Detecting early breast cancer by the combination of five serum microRNAs and its possibility of prediction of pathological complete response in neoadjuvant chemotherapy (Shimomura et al, 2017) | wrong intervention - not looking at the process of IC |
| 234 | Simon-2016-Interactive multimedia consent for biobanking: A randomized trial (Simon et al, 2016) | wrong study design |
| 235 | Sin-2017-What factors influence successful recruitment of siblings of individuals with first episode psychosis to e-health interventions? A qualitative study (Sin et al, 2017) | wrong intervention - not looking at the process of IC |
| 236 | Singer-2010-Communicating disclosure risk in informed consent statements (Singer & Couper, 2010) | wrong study design |
| 237 | Singer-1993-Public opinion regarding consent to treatment (Singer et al, 1993) | wrong intervention - not looking at the process of IC |
| 238 | Siu-2016-Multimedia in the informed consent process for endoscopic sinus surgery: A randomized control trial (Siu et al, 2016) | wrong study design |
| 239 | Skelton-2020-Electronic consenting for conducting research remotely: A review of current practice and key recommendations for using e-consenting (Skelton et al, 2020) | wrong study design |
| 240 | Song-2017-Informed consent for intravenous contrast administration in the emergency department: understanding and satisfaction among patients using the video-assisted vs. traditional methods (Song et al, 2017) | wrong study design |
| 241 | Sonne-2013-Development and pilot testing of a video-assisted informed consent process (Sonne et al, 2013) | wrong study design |
| 242 | Spencer-2015-Using a Multimedia Presentation to Enhance Informed Consent in a Pediatric Emergency Department (Spencer et al, 2015) | wrong study design |
| 243 | Srai-2013-Assessment of the effect of combined multimedia and verbal information vs verbal information alone on anxiety levels before bond-up in adolescent orthodontic patients: a single-center randomized controlled trial (Srai et al, 2013) | wrong study design |
| 244 | Suzuki-2014-The effect of information delivery method on patient comprehension and preference for ultrasound-guided intravenous versus intraosseous access (Suzuki et al, 2014) | wrong intervention - not looking at the process of IC |
| 245 | Tait-2014-Enhancing patient understanding of medical procedures: Evaluation of an interactive multimedia program with in-line exercises (Tait et al, 2014) | wrong study design |
| 246 | Tait-2015-Using digital multimedia to improve parents' and children's understanding of clinical trials (Tait et al, 2015) | wrong study design |
| 247 | Talavera-2016-Levels of participants satisfaction with initial contact and examination visit: The hispanic community health study/study of latinos (HCHS/SOL) (Talavera et al, 2016) | wrong study design |
| 248 | Taylor-2013-Decision making in prostate cancer screening using decision aids vs usual care: a randomized clinical trial (Taylor et al, 2013) | wrong study design |
| 249 | Thornton-2019-Comparing web-based video interventions to enhance university student willingness to donate organs: A randomized controlled trial (Thornton et al, 2019) | wrong study design |
| 250 | Thornton-2016-Effects of a Video on Organ Donation Consent Among Primary Care Patients: A Randomized Controlled Trial (Thornton et al, 2016) | wrong study design |
| 251 | Tipotsch-2016-Effect of a multimedia-assisted informed consent procedure on the information gain, satisfaction, and anxiety of cataract surgery patients (Tipotsch-Maca et al, 2016) | wrong study design |
| 252 | Truong-2020-Multimedia in improving informed consent for caesarean section: A randomised controlled trial (Truong et al, 2020) | wrong study design |
| 253 | Valenza-2008-SmartConsent: a computerized informed consent for dental patients (Valenza et al, 2008) | without full text |
| 254 | Schoenberger-2009-Managing study participant recruitment sites: Methods for optimizing subject recruitment and retention through private practice offices (Schoenberger & Ruetsch, 2009) | wrong intervention - not looking at the process of IC |
| 255 | Vangura-2010-English and spanish video-assisted informed consent for labor epidural: A comparative trial (Vangura & Mordis, 2010) | without full text |
| 256 | Veldhuijzen-2019-E-Patient Counseling Trial (E-Paco): Computer Based Patient Education Is Non-Inferior to Nurse Counseling Prior to Colonoscopy, a Multicenter Randomized Controlled Trial (Veldhuijzen et al, 2019) | duplicates manually removed |
| 257 | Veldhuijzen-2019-E-Patient Counseling Trial (E-PACO): Computer Based Education versus Nurse Counseling for Patients to Prepare for Colonoscopy (Veldhuijzen et al, 2019) | wrong study design |
| 258 | Villanueva-2018-Improving informed consent in cardiac surgery by enhancing preoperative education (Villanueva et al, 2018) | wrong study design |
| 259 | Vo-2018-A randomized trial of multimedia-facilitated informed consent for cataract surgery (Vo et al, 2018) | wrong study design |
| 260 | Wanzer-2010-Enhancing the "Informed" in informed consent: A pilot test of a multimedia presentation (Wanzer et al, 2010) | wrong study design |
| 261 | Warriner-2016-A pragmatic randomized trial comparing tablet computer informed consent to traditional paper-based methods for an osteoporosis study (Warriner et al, 2016) | wrong study design |
| 262 | Wasserman-2013-Adequacy of traditional verbal informed consent versus patient-directed standardized written consent (Wasserman et al, 2013) | wrong intervention - informed consent without electronic component |
| 263 | Westreich-1995-Patient knowledge about electroconvulsive therapy: Effect of an informational video (Westreich et al, 1995) | wrong intervention - not looking at the process of IC |
| 264 | Wilhelm-2009-Extended preoperative patient education using a multimedia DVD-impact on patients receiving a laparoscopic cholecystectomy: A randomised controlled trial (Wilhelm et al, 2009) | wrong intervention - not looking at the process of IC |
| 265 | Winter-2016-The use of portable video media vs standard verbal communication in the urological consent process: a multicentre, randomised controlled, crossover trial (Winter et al, 2016) | wrong study design |
| 266 | Wirshing-2005-A videotape intervention to enhance the informed consent process for medical and psychiatric treatment research (Wirshing et al, 2005) | wrong study design |
| 267 | Wollinger-2012-Computer-based tutorial to enhance the quality and efficiency of the informed-consent process for cataract surgery (Wollinger et al, 2012) | wrong study design |
| 268 | Wysocki-2012-Impact of preoperative information on anxiety and disease-related knowledge in women undergoing mastectomy for breast cancer : A randomized clinical trial (Wysocki et al, 2012) | wrong intervention - not looking at the process of IC |
| 269 | Xia-2019-Video education can improve awareness of risks for patients undergoing endoscopic retrograde cholangiopancreatography: A randomized trial (Xia et al, 2019) | wrong study design |
| 270 | Yeh-2017-Using media to improve the informed consent process for youth undergoing pediatric endoscopy and their parents (Yeh et al, 2017) | wrong study design |
| 271 | Yin-2015-Web-based education prior to knee arthroscopy enhances informed consent and patient knowledge recall: A prospective, randomized controlled study (Yin et al, 2015) | wrong study design |
| 272 | Yoon-2019-Personalized 3D-Printed Model for Informed Consent for Stage I Lung Cancer: A Randomized Pilot Trial (Yoon et al, 2019) | wrong study design |
| 273 | Yu-2018-Incorporating patient information preferences into informed consent (Yu et al, 2018) | wrong study design |
| 274 | Zevin-2019-A Novel Digital Approach to Informed Consent for Roux-en-Y Gastric Bypass: A Randomized Controlled Trial (Zevin et al, 2019) | wrong study design |
