# Supplementary material for: Electronic informed consent: effects on enrolment, practical and economic benefits, challenges, and drawbacks—a systematic review of studies within randomized controlled trials
Source: Trials. 2023 Feb 21;24:127. doi: 10.1186/s13063-022-06959-6 (PMC9942032; doi:10.1186/s13063-022-06959-6)
Supplement: Supplementary file 5 — Additional file 5: Appendix 4. Expanded results table of all included studies. Table containing full results of included studies. [file 13063_2022_6959_MOESM5_ESM.docx]

Appendix 4: Expanded results table of all included studies

| **General Information** |  |
| --- | --- |
| Lead Author | **Afolabi, Muhammed Olanrewaju**^47^ |
| Country in which the study was conducted | Gambia |
| **Characteristics of included studies** |  |
| *Methods* |  |
| Design of the study within RCT | Clinical Trial |
| Health topic | Anti-malarial treatment |
| Health care setting | Outpatient |
| Publication year | 2015 |
| *Participants* |  |
| Population description | Adults with asymptomatic malaria. Majority of participants age<50, more than half women with no formal education or previous clinical trial participation |
| Inclusion criteria | 1) Individuals eligible for PRINOGAM trial 2) ≥18 years or older  3) Speak and understand one of the three major Gambian languages (i.e., Mandinka, Fula, or Wolof)  4) No obvious communication, visual or cognitive impairments |
| Exclusion criteria | See inclusion |
| Method of recruitment of participation | Face to face |
| Total number of participants | 311 |
| Type of intervention | Video information |
| Type of comparator | Acceptable written versions of the local languages. In their absence, the study’s principal investigator trained field staff, who were native speakers of the major local languages, on the correct interpretation of the English version of the participants’ information sheet. Subsequently, the study information was presented verbally to prospective participants during discussions on IC. Consent was given by either signing or thumb-printing the consent form |
| Outcomes | Primary outcome: comprehension of consent information on day 0  Secondary outcomes: comprehension on days 7, 14, 21 and 28 |
| Method of outcomes assessment | Comprehension questionnaire administered using laptop computers by trained interviewers who entered participants’ responses to each question. Responses automatically recorded in the questionnaire computer database. |
| Findings | Significant improvement in comprehension on Day 0 in intervention group (64%) compared to (40%) in the control arm p=0.042. Comprehension remained significant at subsequent follow up visits. |
| Notes | The improvement in comprehension intervention group remained significant across all time points up to 4 weeks compared to the control. Might be a good way for participants with no formal education or previous trial participation in developing countries. |

| **General Information** |  |
| --- | --- |
| Lead Author | **Barrera, Alinne Z.**^50^ |
| Country in which the study was conducted | Worldwide, 23 countries. |
| **Characteristics of included studies** |  |
| *Methods* |  |
| Design of the study within RCT | Observational |
| Health topic | Postpartum depression |
| Health care setting | Community and remote |
| Publication year | 2016 |
| *Participants* |  |
| Population description | 1,179 women included. Participants’ mean age: 27.6 years (SD = 5.6 years); half (52.5%) married or in a relationship; the majority (81.5%) had a university level education; (60%) currently not employed. Ethnic backgroung (81.9%) latino, (86.6%) completed the study in Spanish |
| Inclusion criteria | Pregnant women over 18 interested in the website for personal use |
| Exclusion criteria | Currently meeting diagnosis of major depressive episode |
| Method of recruitment of participation | Online via Google Ads 'sponsored links', email requested & presented with Informed Consent Form |
| Total number of participants | 1179 |
| Type of intervention | Automated online IC |
| Type of comparator | None |
| Outcomes | Understanding of the information: purpose, benefits, and risks |
| Method of outcomes assessment | Multiple-choice items about participants’ understanding of the purpose of the study, the voluntary approach to research participation, potential risks, and potential benefits to participation |
| Findings | 86% understood the purpose of study correctly, 56% identified some or all of the risks correctly, 21% incorrectly identified that there were no risks associated with study |
| Notes | None |

| **General Information** |  |
| --- | --- |
| Lead Author | **Bobb, Morgan**^41^ |
| Country in which the study was conducted | United States |
| **Characteristics of included studies** |  |
| *Methods* |  |
| Design of the study within RCT | Clinical Trial |
| Health topic | Hospital-acquired pneumonia prevention |
| Health care setting | Hospital, emergency department |
| Publication year | 2016 |
| *Participants* |  |
| Population description | From 131 patients randomized, 61(49%) participants were male, 62 (50%) participants lived in nonurban areas (20), and the mean age was 55 years. Groups were well balanced with regard to diagnoses, illness severity, and demographic factors |
| Inclusion criteria | Adult (age≥18 years) patients being treated in a 60,000-visit emergency department at a Midwestern academic medical center between May 2015 and August 2015 were eligible for participation in the study if they were expected to be admitted to the hospital |
| Exclusion criteria | Pregnant women, prisoners, patients with an allergy to chlorhexidine documented in the medical record, non-English speakers, and those with impaired ability to provide IC |
| Method of recruitment of participation | Face to face |
| Total number of participants | 131 |
| Type of intervention | Telemedicine as an aid to paper consent |
| Type of comparator | IC document |
| Outcomes | Primary outcome: Comprehension of research consent  Secondary outomes: Parent trial accrual rate and qualitative survey data |
| Method of outcomes assessment | Quality of IC (QuIC) instrument completed after conclusion of the remainder of parent trial procedures (part A: objective understanding, part B: subjective understanding, part C: qualitative survey information about the use of telemedicine) |
| Findings | No significant differences in objective understanding of research IC in participants consented via telemedicine vs face to face (difference=0.0 points, 95% CI= -3.0 to 3.0). (QuIC scores 74.4 ±_8.1 vs. 74.4_± 6.9 on a 100-point scale, p = 0.999). No significant differences for subjective consent (difference=1.8points, 95% CI= -0.4 to 7.1) No statistically significant difference in parent trial accrual rates (56% vs. 69%, p=0.142) No significant barriers identified in qualitative survey |
| Notes |  |

| **General Information** |  |
| --- | --- |
| Lead Author | **Ditai, James**^48^ |
| Country in which the study was conducted | Uganda |
| **Characteristics of included studies** |  |
| *Methods* |  |
| Design of the study within RCT | Observational |
| Health topic | Neonatal sepsis |
| Health care setting | Community |
| Publication year | 2018 |
| *Participants* |  |
| Population description | 30 women included. A third (11/30) of the women did not know their exact dates of birth. Most of the women (77%, 23/30) were married while two-thirds were either unemployed or peasant farmers. Half of the women had no formal education or did not complete primary education, which could be an indicator of the level of literacy amongst this population. All the women lived with one or more family members. More than half (60%, 18/30) had family members present while the consent information was being read, presented or shown |
| Inclusion criteria | Confirmed pregnant women with gestation of 34 weeks or more and able to speak or understand English or Lumasaba (the local language) |
| Exclusion criteria | Women who had already been recruited into the pilot trial before this nested study |
| Method of recruitment of participation | Face to face |
| Total number of participants | 30 |
| Type of intervention | Slide show using illustrated text on a flip chart or video |
| Type of comparator | Standard researcher-read patient information sheet |
| Outcomes | Understanding and recalling information about the BabyGel pilot trial 48 hs using the modified Quality of IC form |
| Method of outcomes assessment | Modified Quality of IC form (QuIC) to measure subjetive and objective comprehension and semi-structured interview |
| Findings | High understanding of the study for all three consent models. No statistically significant difference between the slide-show message (mean 4.7; standard deviation, SD 0.47; range 4–5), video message (mean 4.9; SD 0.33; range 4–5) and standard method (mean 4.5; SD 0.53; range 4–5; all one-way ANOVA, p = 0.190). The slide-show message resulted in the most objective understanding of question items with the highest average QuIC score of 100 points. Most women (63%, 19/30) preferred the slide-show message, compared with 17% (5/30) and 20% (6/30) for the standard and video messages, respectively |
| Notes | An analysis of the semi-structured interviews revealed three themes: pictorial illustrations aid understanding, logical progression of information, and ease of understanding for women who are illiterate |

| **General Information** |  |
| --- | --- |
| Lead Author | **Dobscha, Steven**^42^ |
| Country in which the study was conducted | United States |
| **Characteristics of included studies** |  |
| *Methods* |  |
| Design of the study within RCT | Observational |
| Health topic | Depression |
| Health care setting | Hospital clinics and remote community-based clinics |
| Publication year | 2005 |
| *Participants* |  |
| Population description | From 400 participants, 369 were interviewed in person and 31 through video conference. No statistically significant differences between both groups: 87% to 93% were male. Mean age varied from 57 to 59. No differences reported in mean depression or posttraumatic stress disorder scores |
| Inclusion criteria | Patients who were due to see primary care providers within a month, and whose primary care providers (n=44) were participating in Depression in Primary Care |
| Exclusion criteria | Patients who had received treatment from a mental health care clinician within the prior 6-month period, or had Alzheimer’s disease, cognitive problems, psychotic symptoms, or terminal illness noted in their medical record |
| Method of recruitment of participation | Phone |
| Total number of participants | 31 |
| Type of intervention | Videoconferencing |
| Type of comparator | IC document |
| Outcomes | Participant satisfaction with various aspects of the interview e.g. technical, accessibility, convenience, interviewer's ability to understand responses, participant understanding |
| Method of outcomes assessment | 12-item mail survey |
| Findings | No significant problems with the process of interviewing and obtaining IC by videoconferencing. High degree of satisfaction with the interviews. Participants expressed willingness to recommend videoconferencing to others |
| Notes | Participants gave somewhat lower marks for their comfort with the session relative to an in-person interview, as well as their ability to hear. When asked what they would change about the video, two categories were most frequently reported. Several participants stated that the 5-min video was too long (“This is getting a little long”; and “The length [shorten it].”). Other participants thought that the disclosure about risks for this minimal risk study should be changed. No differences were observed between the Patient Health Questionnaire depression scores of videoconferencing and in-person participants, and there was no significant difference in the 6-month rate of loss to follow-up in the randomized trial. |

| **General Information** |  |
| --- | --- |
| Lead Author | **Nogueira, Raul G.**^39^ |
| Country in which the study was conducted | United States |
| **Characteristics of included studies** |  |
| *Methods* |  |
| Design of the study within RCT | Observational |
| Health topic | Stroke |
| Health care setting | Hospital |
| Publication year | 2017 |
| *Participants* |  |
| Population description | Six surrogates were e-Consented in the DAWN trial, 2 excluded from trials at screening, n=4. Mean age 73. Similar National Institutes of Health Stroke Scale (NIHSS) in e-Consented compared to conventional consent (n = 25) |
| Inclusion criteria | Patients eligible for the DAWN and ARISE II trial |
| Exclusion criteria | See inclusion |
| Method of recruitment of participation | Phone, Research Electronic Data Capture (REDCap) |
| Total number of participants | 4 |
| Type of intervention | For DAWN trial: all 3 components of IC electronic. The method for ARISE-I is presumed the same. |
| Type of comparator | Traditional written IC |
| Outcomes | To describe the first experience with electronic informed consenting |
| Method of outcomes assessment | Data collected and managed using REDCap |
| Findings | Time from door-to-randomization was decreased with e-Consenting (28±9 versus 57±24 minutes; P=0.002) streamlining the consenting process |
| Notes | None of the e-Consented surrogates had any reservations about the method used for consenting |

| **General Information** |  |
| --- | --- |
| Lead Author | **Jolly, Kate**^45^ |
| Country in which the study was conducted | United Kingdom |
| **Characteristics of included studies** |  |
| *Methods* |  |
| Design of the study within RCT | Clinical Trial |
| Health topic | COPD |
| Health care setting | Outpatient |
| Publication year | 2019 |
| *Participants* |  |
| Population description | Population was mostly male, with a mean age of 70 years, with limited educational qualifications and with most retired from work. Authors were unable to assess whether those recruited by multimedia differed in characteristics from those who were recruited using conventional methods. |
| Inclusion criteria | 1) age 18+ years 2) on the practice COPD register 3) experience mild dyspnoea (Medical ResearchCouncil (MRC) grades 1 or 2), 4) FEV1/FVC < 0.7 after post-bronchodilator spirometry |
| Exclusion criteria | See inclusion |
| Method of recruitment of participation | Letter and telephone |
| Total number of participants | 4214 |
| Type of intervention | Standard printed patient information materials with access to a multimedia information resource |
| Type of comparator | Standard printed patient information materials |
| Outcomes | Primary outcome: recruitment rate, defined as the proportion of patients actually recruited to the host trial following an invitation and randomised to each group Secondary outcomes: numbers responding to the trial invitation, as well as 6- and 12-months’ retention rates |
| Method of outcomes assessment | Number of patients recruited to host trial and responding invitation |
| Findings | No effects on recruitment: 9.6% recruited of those receiving standard printed patient information materials and access to the multimedia information resource compared to 10.8% in those receiving standard printed materials alone (odds ratio (OR) = 0.844, 95% confidence interval (CI) 0.58 to 1.22). No effects on the proportion of people responding to the invitation (OR = 1.02, 95% CI 0.79 to 1.33) or retention in the trial at 6 (ORs 0.84, 95% CI 0.57 to 1.22) and 12 months after randomisation (ORs 0.80, 95% CI 0.54 to 1.18), respectively. |
| Notes | Participants in the e-IC group were asked about other study-specific details such as what the study was about, the benefits of the study, and how a participant was assigned to one of the three study groups. All of the participants were able to recall accurate and detailed responses about the study. These responses indicated that study-specific details were understood. |

| **General Information** |  |
| --- | --- |
| Lead Author | **Lurie, Jon D.**^43^ |
| Country in which the study was conducted | United States |
| **Characteristics of included studies** |  |
| *Methods* |  |
| Design of the study within RCT | Observational |
| Health topic | Spine surgery |
| Health care setting | Outpatient |
| Publication year | 2011 |
| *Participants* |  |
| Population description | From 2505 patients, 2151 (86%) watched the video. Population stratified by diagnosis (IDH vs. SPS/DS). IDH patients shifting toward surgery compared to those shifting to non-operative care: worse baseline bodily pain (SF-36 Bodily Pain score 24 vs. 30; p=0.003), physical function (SF-36 Physical Function score 33 vs. 47; p<0.001), disability (Oswestry Disability Index 53 vs. 43; p<0.001), and greater dissatisfaction with their symptoms (very dissatisfied 85% vs. 71%; p=0.006). SPS/DS patients had worse symptoms, lower function and greater disability among those who shifted their preference toward surgery; more likely to be female; to be receiving disability compensation; to have previously received an epidural steroid injection; and were less likely to have diabetes mellitus. Race, ethnicity, educational attainment, marital status, work status, BMI, smoking status, duration of symptoms, leg pain bothersomeness, SF-36 Mental component summary score, prior physical therapy, and baseline symptom trajectory did not significantly differ (IDH mean age from 41.2-42, SPS mean age from 65.1-67.1, IDH female gender 38-46%, SPS female gender 42-52%) |
| Inclusion criteria | Over 18 years old with a clinical diagnosis of either intervertebral discherniation (IDH) or spinal stenosis (SPS) - with or without degenerative spondylolisthesis(DS) - that was confirmed by imaging. All participants had had symptoms for a minimum of 6 weeks in the IDH group and 12 weeks in the SPS/DS groups and were deemed surgical candidates by the enrolling surgeon |
| Exclusion criteria | Cauda equina syndrome, progressive neurological deficit, malignancy, significant deformity, prior back surgery and other established contraindications to elective surgery |
| Method of recruitment of participation | Face to face |
| Total number of participants | 2505 |
| Type of intervention | Evidence-based video decision aid as part of their IC process |
| Type of comparator | Before and after viewing video |
| Outcomes | Changes in treatment preference after watching a video decision aid as part of an IC process |
| Method of outcomes assessment | Patients identified as candidates for the study answered the question “What is your current preference for how to treat your spine-related problem?” on a 5-point scale. After enrolment patient preferences (including those of non-watchers) were again assessed using the same scale |
| Findings | Of the 2505 patients, 86% (n=2151) watched the video and 14% (n=354) did not. Watchers shifted their preference more often than non-watchers (37.9% vs. 20.8%, p < 0.0001) and more often demonstrated a strengthened preference (26.2% vs. 11.1%, p < 0.0001). Among the 806 patients whose preference shifted after watching the video, 55% shifted toward surgery(p=0.003). Among the 617 who started with no preference, after the video 27% preferred non-operative care, 22% preferred surgery, and 51% remained uncertain |
| Notes | Before and after comparison with intervention. All patients were given an evidence-based video decision aid as part of their IC process; there were two separate video decision aids, one for the IDH group and another for the SPS and DS groups. A small percentage of the patients chose not to watch the video |

| **General Information** |  |
| --- | --- |
| Lead Author | **Mattock, Holly**^46^ |
| Country in which the study was conducted | United Kingdom |
| **Characteristics of included studies** |  |
| *Methods* |  |
| Design of the study within RCT | Clinical Trial |
| Health topic | Prevention of behavioral problems in young children |
| Health care setting | Outpatient |
| Publication year | 2020 |
| *Participants* |  |
| Population description | 107 were eligible to be included in the SWAT sample. Fourteen participants were excluded as they had not provided an email address. Of the 107 participants, most were identified as ‘White’ (79%), biological mothers (93%) and educated to postgraduate level (47%). The mean parent age was 33.59 years (N = 105, SD = 5.4), and the mean child age was 21.9 months (SD = 0.5); slightly more male than female children participated (58% vs. 41%) |
| Inclusion criteria | Parents aged≥18 years, child aged between approximately 12 and 36months, a score in the top 20% on population norms for child behaviour problems using the Strengths and Difficulties Questionnaire (SDQ; Goodman) and written IC. Additional eligibility criteria required for SWAT inclusion was that participants needed to provide a valid email address |
| Exclusion criteria | 1) Participants who only provided a telephone number as contact details  2) Participants who were already participating in the HS,HS trial |
| Method of recruitment of participation | Face to face and mailshots |
| Total number of participants | 107 |
| Type of intervention | Informational video clip as an aid to patient information sheet |
| Type of comparator | Written Patient Information Sheet |
| Outcomes | Primary outcome: proportion of participants who consented to take part in the main trial based upon their SWAT allocation  Secondary outcome: participant and researcher attitudes towards method of recruitment via brief structured interviews |
| Method of outcomes assessment | Number of participants consented in main trial and brief structured interviews |
| Findings | 10/56 video clip vs 26/51 PIS consented. Video condition did not increase the odds of recruitment into the trial, such that those in the video condition were significantly less likely to participate in the main trial (OR = 0.253, CI = 0.104–0.618, p = 0.003) |
| Notes | 5 participants in video and 12 in PIS were interviewed. Irrespective of condition, all participants commented positively on how useful they found the introductory information, stating it was easy to understand and informative but also stated they had further questions that needed discussing over the phone. Participants in video group framed the material as introductory, whilst those in PIS described it as comprehensive. Key finding from both researcher interviews was that the initial email contact increased participants’ receptivity to the study team and engagement in the trial. Researchers found that participants had often not watched the video clip or viewed a portion of it, although those who had viewed it were reported as having a better understanding of randomisation. The advantage of participants seeing images of the research team and activities in the video clip was also highlighted. |

| **General Information** |  |
| --- | --- |
| Lead Author | **Rothwell, Erin**^40^ |
| Country in which the study was conducted | United States |
| **Characteristics of included studies** |  |
| *Methods* |  |
| Design of the study within RCT | Clinical Trial |
| Health topic | Prenatal education |
| Health care setting | Outpatient |
| Publication year | 2014 |
| *Participants* |  |
| Population description | All participants were female and 61% had given birth before. Of those who had given birth before, 31% had given birth to one child and 19.4% had given birth to two or more children. 93.55% were married or living together, 90.32% were non-Hispanic, 41.94% were educated at a Bachelor degree |
| Inclusion criteria | English speaking women with full-term pregnancies who gave birth with normal birth outcomes |
| Exclusion criteria | See inclusion |
| Method of recruitment of participation | Face to face |
| Total number of participants | 62 |
| Type of intervention | Video presentation on an iPad as an aid to paper consent form |
| Type of comparator | Paper IC form |
| Outcomes | Participant comprehension of the study - specific details |
| Method of outcomes assessment | 14-item survey about the consent process and participants in electronic group underwent telephone interview about the video |
| Findings | Participants on video group had better understanding in certain aspects of the study but not others compared to the control group. E.g. better understanding in the purpose of the study, alternatives to participation, and who to contact if they had questions or concerns about the study |
| Notes | Some survey items had higher mean scores for the paper-based consent group. These items focused on what the researchers were trying to find in this study, which education approaches were new, benefits, risks, and voluntary participation |

| **General Information** |  |
| --- | --- |
| Lead Author | **Swain, Sandra**^44^ |
| Country in which the study was conducted | United States |
| **Characteristics of included studies** |  |
| *Methods* |  |
| Design of the study within RCT | Observational |
| Health topic | Breast cancer |
| Health care setting | Hospital |
| Publication year | 2017 |
| *Participants* |  |
| Population description | Black breast cancer patients. Mean age of the participants was 59 years, 74.5% had stage I-III disease, 29% were married, 83.5% had one or more children, and 29% attended some college or technical school |
| Inclusion criteria | Breast cancer patients with Stage I-III, if diagnosed within previous 6 months, or metastatic disease who self-identified as black or African American |
| Exclusion criteria | See inclusion |
| Method of recruitment of participation | Face to face |
| Total number of participants | 200 |
| Type of intervention | Educational video |
| Type of comparator | None |
| Outcomes | Primary outcome: enrolment to a therapeutic clinical trial  Secondary outcome: to explore the capacity of the video to influence black breast cancer patients’(a) intentions to participate in a CT; and, (b) attitudes towards CTs |
| Method of outcomes assessment | Patients who signed consent regardless if they enrolled in a trial. Attitudes and Intention to Enroll in Therapeutic Clinical Trials (AIET) questionnaire |
| Findings | 200 patients that participated, 39 (19.5%) patients signed consent to participate in a therapeutic clinical trial; 27 (13.5%) patients enrolled (7.5% increase from baseline comparison of 6% clinical trial enrolment rate in black cancer patients (p< .001)). Pre-test versus post-test assessment showed an increase by 14% in proportion of patients expressing likelihood to enroll in a therapeutic trial following the intervention (p< .001). Among 31 AIET items, 25 (81%) showed statistically significant and positive change post-intervention. |
| Notes | The video is a component of ICF as it is a way to enhance delivery of information about enrolling into a clinical trial |

| **General Information** |  |
| --- | --- |
| Lead Author | **Weston, Julie**^49^ |
| Country in which the study was conducted | Canada |
| **Characteristics of included studies** |  |
| *Methods* |  |
| Design of the study within RCT | Clinical Trial |
| Health topic | Obstetric Prelabour Rupture of Membranes |
| Health care setting | Outpatient and clinics |
| Publication year | 1997 |
| *Participants* |  |
| Population description | Baseline characteristics similar between video and control group: median maternal age varied from 31.4 to 31.8, median gestation in weeks varied from 25 to 27.3 and 40 to 42% had achieved a college degree or higher |
| Inclusion criteria | English speaking, with 20-32 weeks gestation (gestational age group that was ineligible for the Term PROM Study) |
| Exclusion criteria | Women who had previously watched the Term PROM video |
| Method of recruitment of participation | Face to face |
| Total number of participants | 90 |
| Type of intervention | Information video |
| Type of comparator | Written IC |
| Outcomes | Primary outcome: willingness for future participation in the Term PROM Study should they become eligible  Secondary outcomes: women’s views on the importance of the Term PROM Study, knowledge of the study protocol, and term prelabour rupture of the membranes |
| Method of outcomes assessment | Questionnaire |
| Findings | More women who watched the video thought they would consent to the study (x2 = 6.3; df = 1: P = 0.01). No differences in knowledge about the perinatal trial were found initially, but 2-4 weeks later more knowledge had been retained by women who had watched the video (x” = 6.7; df = 1; P = O.OI) |
| Notes | None |
