# Supplementary material for: Electronic informed consent: effects on enrolment, practical and economic benefits, challenges, and drawbacks—a systematic review of studies within randomized controlled trials
Source: Trials. 2023 Feb 21;24:127. doi: 10.1186/s13063-022-06959-6 (PMC9942032; doi:10.1186/s13063-022-06959-6)
Supplement: Supplementary file 6 — Additional file 6: Appendix 5. Cochrane risk of bias table of included RCT studies. Risk of bias with full explanation for included RCT. [file 13063_2022_6959_MOESM6_ESM.docx]

Appendix 5: Cochrane risk of bias table of included RCT studies

**Afolabi 2015** ^47^

| **Bias** | **Authors' judgement** | **Support for judgement** |
| --- | --- | --- |
| Random sequence generation (selection bias) | Low risk | Computerised block randomisation & stratification |
| Allocation concealment (selection bias) | High risk | No information provided in sufficient detail to explain efforts to conceal the allocation sequence |
| Blinding of participants and personnel (performance bias) | High risk | No blinding |
| Blinding of outcome assessment (detection bias) | High risk | No blinding |
| Incomplete outcome data (attrition bias) | Low risk | Reasons for not completing the study were specified, 26 refused to take part (7.5%) in the study of the multi-media IC tool as they did not have time to wait because of domestic demands, 10 participants (2.9%) insisted on using the multimedia tool without going through randomization. After excluding these 36 participants, 311 were enrolled in the study and included in final analysis |
| Selective reporting (reporting bias) | Low risk | A matrix was created by review author and verified that there was a complete reporting of all predefined, or clinically relevant and reasonably expected outcomes |
| Other bias | Unclear risk | The study was supported by a grant from the European and Developing Countries Clinical Trials Partnership. Nuala Mc-Grath was supported by a Wellcome Trust Fellowship and Neal Alexander received support from the United Kingdom Medical Research Council and Department for Interna-tional Development |

**Bobb 2016** ^41^

| **Bias** | **Authors' judgement** | **Support for judgement** |
| --- | --- | --- |
| Random sequence generation (selection bias) | Low risk | Patients randomized using 1:1 allocation ratio. Randomization conducted using block randomization with block sizes of 4 |
| Allocation concealment (selection bias) | Low risk | Randomization code concealed in sequentially numbered opaque sealed envelopes |
| Blinding of participants and personnel (performance bias) | High risk | No blinding |
| Blinding of outcome assessment (detection bias) | Unclear risk | The scoring of surveys for the interim analysis was performed by an independent research assistant not affiliated with the study |
| Incomplete outcome data (attrition bias) | Unclear risk | A total of 131 participants were randomized but only 101 quality of IC (QuIC) completed. Report does not explain why the rest were not obtained. |
| Selective reporting (reporting bias) | Low risk | A matrix was created by review author and verified that there was a complete reporting of all predefined, or clinically relevant and reasonably expected outcomes |
| Other bias | Unclear risk | Dr. Mohr is supported by grants from the Emergency Medicine Foundation and the U.S. Department of Health and Human Services Health Resources and Services Administration. This study was supported by the University of Iowa Department of Emergency Medicine and the University of Iowa Carver College of Medicine (NIH training grant 2T35HL007485-36). The University of Iowa Department of Emergency Medicine sponsors a rural emergency telemedicine network |

**Jolly 2019** ^45^

| **Bias** | **Authors' judgement** | **Support for judgement** |
| --- | --- | --- |
| Random sequence generation (selection bias) | Low risk | Practices (cluster level) in host trial were randomised using stratified (by area – Birmingham, Oxford, Manchester, and Stoke-on-Trent) block randomisation (ratio 1:1, with three varying block sizes selected randomly by the computer) to access to the multimedia information resource or only the printed patient information sheet |
| Allocation concealment (selection bias) | Low risk | Allocation sequence was generated centrally by VM (who had no other involvement in the running of the host trial) using the ‘ralloc’ command in Stata |
| Blinding of participants and personnel (performance bias) | Unclear risk | Although IC was gained from patients in the host trial, patients were not aware that they were being randomised within the SWAT and no formal consent was taken |
| Blinding of outcome assessment (detection bias) | High risk | No blinding |
| Incomplete outcome data (attrition bias) | Unclear risk | Reasons for lost to follow up were not stated. |
| Selective reporting (reporting bias) | Low risk | A matrix was created by review author and verified that there was a complete reporting of all predefined, or clinically relevant and reasonably expected outcomes |
| Other bias | Low risk |  |

**Mattock 2020** ^46^

| **Bias** | **Authors' judgement** | **Support for judgement** |
| --- | --- | --- |
| Random sequence generation (selection bias) | High risk | Randomisation list for the initial contact prepared by trial statistician. Allocation released weekly to research team |
| Allocation concealment (selection bias) | High risk | Participants allocated based on the week they returned their screening questionnaire. Research team not blinded to group allocation |
| Blinding of participants and personnel (performance bias) | High risk | No blinding |
| Blinding of outcome assessment (detection bias) | High risk | No blinding |
| Incomplete outcome data (attrition bias) | Unclear risk | Reasons for qualitative results were specified (Qualitative feedback: for practical reasons only 17 participants were invited to interview). Reasons for not participating in study were not stated (Quantitative: Of the 50 participants given the video clip, 10 (20%) consented to take part in the trial, compared with 26 (51%) of 51 participants given information only via the traditional PIS). |
| Selective reporting (reporting bias) | Low risk | A matrix was created by review author and verified that there was a complete reporting of all predefined, or clinically relevant and reasonably expected outcomes |
| Other bias | Unclear risk | The study was supported by the National Institute for Health Research Imperial Biomedical Research Centre and Imperial Clinical Trials Unit based at Imperial College Healthcare NHS Trust and Imperial College London |

**Rothwell 2014** ^40^

| **Bias** | **Authors' judgement** | **Support for judgement** |
| --- | --- | --- |
| Random sequence generation (selection bias) | High risk | Not stated |
| Allocation concealment (selection bias) | High risk | Not stated |
| Blinding of participants and personnel (performance bias) | High risk | No blinding |
| Blinding of outcome assessment (detection bias) | High risk | No blinding |
| Incomplete outcome data (attrition bias) | Unclear risk | Outcomes are not clearly stated in the report. |
| Selective reporting (reporting bias) | Unclear risk | Outcomes are not clearly stated in the report. |
| Other bias | Unclear risk | The University of Utah Annette Poulson Cumming College of Nursing provided funding for this research |

**Weston 1997** ^49^

| **Bias** | **Authors' judgement** | **Support for judgement** |
| --- | --- | --- |
| Random sequence generation (selection bias) | Low risk | Women randomised video or control group. Randomization list generated using random numbers table and held centrally at the Data Coordinating Centre |
| Allocation concealment (selection bias) | Unclear risk | The study nurse obtained each participant’s allocation assignment by phone from a member of the study staff |
| Blinding of participants and personnel (performance bias) | High risk | No blinding |
| Blinding of outcome assessment (detection bias) | High risk | No blinding |
| Incomplete outcome data (attrition bias) | Unclear risk | Questionnaires were completed by all women at enrolment and by 85 women two to four weeks later. Reasons for not completing the rest of questionnaires were not stated. |
| Selective reporting (reporting bias) | Low risk | A matrix was created by review author and verified that there was a complete reporting of all predefined, or clinically relevant and reasonably expected outcomes |
| Other bias | Low risk |  |
