# Supplementary material for: Electronic informed consent: effects on enrolment, practical and economic benefits, challenges, and drawbacks—a systematic review of studies within randomized controlled trials
Source: Trials. 2023 Feb 21;24:127. doi: 10.1186/s13063-022-06959-6 (PMC9942032; doi:10.1186/s13063-022-06959-6)
Supplement: Supplementary file 9 — Additional file 9: Appendix 8. Quality Assessment. Complete analysis of Risk of Bias in RCT, Case Control and Cohort studies. [file 13063_2022_6959_MOESM9_ESM.docx]

Appendix 8

Quality Assessment

Randomised Controlled Trials

The quality of included studies varied. All six RCTs included in this review (40, 41, 45-47, 49) were assessed using the Cochrane Risk of Bias tool, and overall, they were judged to be at moderate-to-high risk of bias. Graphical summary of the risk of bias of included RCTs are shown in Figure 2 and Figure 3. Assessment of risk of bias using the Cochrane risk of bias table is detailed in Appendix 5.

*Random Sequence Generation*

Four (41, 45, 47, 49) out of six RCTs were assessed as low risk of bias for this domain, as they described the use of a block randomisation. The remaining two RCTs were judged as high risk for not providing an explanation on their sequence generation (40).

*Allocation Concealment*

Half of the RCTs were assessed as high risk of bias for this domain(40, 46, 47), as there was not sufficient information provided in detail to explain efforts to conceal the allocation sequence. One study referred to a list of allocation prepared by the trial statistician, and it was unclear whether trial staff could have foreseen the allocation, which could have introduced selection bias(46). Two RCTs were assessed as low risk of bias as they described adequate allocation concealment. One study was judged as having unclear bias due to insufficient description of the process for concealing the allocation, stating that “study nurse obtained each participant’s allocation assignment by phone from a member of the study staff”(49, pp. 241).

*Blinding of participants and personnel*

Not ensuring participants and personnel are blinded to their allocation of intervention may lead to performance bias. The majority of the RCTs were judged as being at high risk of bias for blinding of participants and personnel. Only one trial was labeled as unclear risk as it simply reported that “patients were not aware that they were being randomised within the SWAT” without giving any further details on how this was achieved(45, pp. 3).

*Blinding of outcome assessment*

Three of the clinical trials contained quantitative primary outcome data (rate of enrolment)(45, 46, 49).The rest of the RCTs covered self-reported outcomes (participant´s comprehension of information, information recalling, willingness for future participation in research, effect on changes in treatment preferences, description of electronic consent experience)which were collected either by researcher or by self-completion of surveys/ questionnaires. Five out of six RCTs were judged as high risk of detection bias as blinding of outcome assessment was not performed. Only one study(41) with self-reported outcome was assessed as unclear risk of bias as it reported that an assistant not affiliated with the study was responsible for the scoring of surveys for the interim analysis.

*Incomplete outcome data*

Only two included RCTs were judged by the review author as being at low risk of attrition bias as they reported4% (47)and 5.6%(49) of missing outcome data. The rest of the RCTs were assessed as unclear risk as reasons for incomplete outcome data were not provided.17/36 completed secondary outcomes were reported in one study (46). Rothwell et al. did not clearly state the study’s primary outcome but reported surveys on participant comprehension were not completed by all participants. Bobb et al. reported that the primary outcome of comprehension of consent was only completed in 101 out of 131 participants but did not provide reasons incomplete outcome data. Jolly et al. did not report reasons for lost to follow up.

*Selective Reporting*

Almost all included RCTs were judged by review author as being at low risk of reporting bias. Although study protocol was not available for review, all trials reported outcomes of interest as pre-specified. One RCT(40) was labelled as unclear risk as outcomes were not clearly defined in the report.

*Other sources of bias*

Reporting and publication bias could not be assessed by funnel plots as stated in the protocol as there were not sufficient RCTs included under a particular comparison. Only four RCTs reported their sources of funding for their research(40, 41, 46, 47).

Case control and Cohort studies

The assessment of risk of bias for non-randomised studies was performed using the CASP checklist (36). Overall, studies were judged as high risk of bias. CASP checklist template states that if the answer to the first two questions is different from “yes”, it is not worth proceeding with the remaining questions, but for the purpose of summarizing the risk of bias for this dissertation, authors analyzed all CASP questions and used the scoring system as detailed in section 4.6.

*The assessment*

All studies were assessed to be high risk of bias, scoring between two and three as there was not sufficient information to perform a detailed assessment. There were a mixture of secondary analyses, case control studies, cohort studies and studies that lacked objective outcome measures.

*Case control*

Five studies(39, 42-44, 50) were case control studies and analyzed under this category. All five studies addressed clearly focused issues: electronic consent for online resources for perinatal women, experiences in stroke trials, effects of videoconferencing in depression management, changes in preferences for spine surgery and the impact of video information on enrolment of black breast cancer patients. The study designs varied from secondary analysis of larger studies (39, 50), to non-randomised, uncontrolled trial(42-43), and case series with comparisons before and after an intervention (44).

The suitability of these observational study designs had to be assessed against the specific research question. While ideally, a randomized controlled trial would be the gold-standard method of assessing the efficacy of an intervention, the authors judged all of the included observational studies to be suitable when assessed against the specific research questions e.g. feasibility or preliminary user experience. All studies had ethics approval.

For all five studies analyzed under case control template, authors judged the recruitment process as unclear and insufficiently detailed. Recruitment sample was selective in most studies, either self-selected or selected by study researcher, thus introducing selection bias. Although study demographics seemed representative of study population, only one study(44)included a power size calculation.

Barrera et al. and Swain et al. did not include a comparator or control. Lurie et al. compared pre- and post- video intervention, Dobscha et al. compared video vs face to face consent and Haussen et al. compared electronic vs written IC.

The exposure was the electronic consent intervention, which differed between studies and not easily measurable.

The majority of outcomes were self-reported so objectivity might have been compromised and inter-rater variability introduced. Surveys were used to measure outcomes in most studies with only one study (39) confirmed that the tool was validated prior to its use. Potential confounding factors were not accounted for in any of the studies that compared two groups(39, 42).

Overall, study results were judged as having unclear risk of bias as there was insufficient information to assess the question and account for every consideration included in the CASP template. Studies reported basic descriptive statistics with no confidence intervals.

Per author reviewer´s opinion, results can help locally. Results of studies by Lurie et al. and Swain et al. reported that their results fitted with other available evidence. The rest of the studies reported limited prior evidence for the topics investigated.

*Cohort study*

Only one cohort study was included in this review (48), which was judged to be high risk of bias. This was a small cohort study (n = 30) with three observational arms, embedded within a clinical trial. The study had a clearly focused question: the impact of using audio-visual presentations on the participant understanding of clinical trial information. The sample was representative of women of low or no literacy in a low-income setting in rural eastern Uganda. Participants were allocated to one of the three consenting models (standard researcher read v slide show v video show) that were in use that week. The consenting model then changed to another after each week. The absence of randomisation meant that there were likely imbalances in the participants’ characteristics. Objective procedures and validated measures were used to assess

outcomes. No confounding factors were adjusted for in the analyses. There was no loss to follow up. Sample size was small which meant that any differences detected between the consenting methods could be due to change alone. This study’s results may be applicable to the specific local population, but it was unclear if results from this study fitted with other available evidence. There were no implications for practice reported either.

**Cochrane Risk of Bias for RCT with reasons**

|  | **Afolabi-2015**^47^ | **Mattoc-2020**^46^ | **Rothwell-2014**^40^ | **Bobb-2016**^41^ | **Jolly-2019**^45^ | **Weston-1997**^49^ |
| --- | --- | --- | --- | --- | --- | --- |
| **Sequence Generation** | Low. Computerised block randomisation & stratification | High. Participants were randomly allocated to receive one of two initial contacts from the research team: video and PIS or PIS alone. A randomisation list for the initial contact was prepared by the trial statistician, and allocation was released weekly to the research team | High. Not stated | Low. Patients were randomized to telemedicine enabled or F2F research consent using a 1:1 allocation ratio. Randomization was conducted using block randomization with block sizes of 4. | Low. To ease the logistics of the trial, practices (cluster level) in the host trial were randomised using stratified (by area – Birmingham, Oxford, Manchester, and Stoke-on-Trent) block randomisation (ratio 1:1, with three varying block sizes selected randomly by the computer) to access to the multimedia information resource or only the printed patient information sheet (i.e. all patients from a particular practice were sent the same invitation letter). | Low. Women were randomised to either watch the Term PROM video (video group) or not (control group). A randomization list was generated using a random numbers table and held centrally at the Data Coordinating Centre. |
| **Allocation Concealment** | High. No information provided in sufficient detail to explain efforts to conceal the allocation sequence. | High. Each participant was allocated to their condition based on the week they returned their screening questionnaire. The research team were not blinded to group allocation | High. Not stated | Low. Randomization code was concealed in sequentially numbered opaque sealed envelopes. | Low. To ensure allocation concealment, the allocation sequence was generated centrally by VM (who had no other involvement in the running of the host trial) using the ‘ralloc’ command in Stata. | Unsure. The study nurse obtained each participant’s allocation assignment by phone from a member of the study staff. |
| **Blinding of participants and personnel** | High. No blinding | High. No blinding | High. No blinding | High. No blinding | Unsure. Although informed consent was gained from patients in the host trial, patients were not aware that they were being randomised within the SWAT and no formal consent was taken. | High. No blinding |
| **Blinding of outcome assessment** | High. No blinding | High. No blinding | High. No blinding | Unsure. The scoring of surveys for the interim analysis was performed by an independent RA not affiliated with the study, such that aggregate survey results were not available to the RA conducting the research. | High. No blinding | High. No blinding |
| **Incomplete outcome data** | Low. Reasons for not completing the study were specified, differentiating the ones which refused to take part and the ones not going through randomization. Of the 347 participants enrolled in the PRINOGAM trial, 26 refused to take part (7.5%) in the study of the multi-media informed consent tool. Most of those who refused said they did not have time to wait because of domestic demands. In addition, 10 participants (2.9%) insisted on using the multimedia tool without going through randomization, most likely because they had heard about the tool through friends or family already enrolled in the study. After excluding these 36 participants, 311 were enrolled in the study and included in final analysis | Unsure. Reasons for qualitative results were specified (Qualitative feedback: Whilst the initial aim was to gain feedback from all SWAT participants who were randomised (n = 36), for practical reasons (e.g. time demands of the assessment) and the importance of not overburdening participants 17 participants were invited to interview. All 17 participants who were approached agreed to interview) but reasons for not participating in study were not stated (Quantitative: Of the 50 participants given the video clip, 10 (20%) consented to take part in the trial, compared with 26 (51%) of 51 participants given information only via the traditional PIS). | Unsure. Outcomes are not clearly stated in the report. | Unsure. Comprehension of research consent (primary outcome) was measured using the modified quality of informed consent (QuIC) instrument. A total of 131 subjects were randomized but only 101 QuIC completed. Report does not explain why the rest were not obtained. | Unsure. The primary outcome was recruitment rate, defined as the proportion of patients actually recruited to the host trial following an invitation and randomised to each group. Analysis was by intention-to-treat. The numbers responding to the trial invitation, as well as 6 and 12months’ retention rates were secondary outcomes. Reasons for lost to follow up were not stated. | Unsure. The study enrolled 90 women between June 28 and December 22, 1994, 42 of whom were allocated to the video group and 48 to the control group. Questionnaires were completed by all women at enrolment and by 85 women two to four weeks later. Reasons for not completing the rest of questionnaires were not stated. |
| **Selective reporting** | Low. A matrix was created by review author and verified that there was a complete reporting of all pre‐defined, or clinically relevant and reasonably expected outcomes | Low. A matrix was created by review author and verified that there was a complete reporting of all pre‐defined, or clinically relevant and reasonably expected outcomes | Unsure. Outcomes are not clearly stated in the report. | Low. A matrix was created by review author and verified that there was a complete reporting of all pre‐defined, or clinically relevant and reasonably expected outcomes | Low. A matrix was created by review author and verified that there was a complete reporting of all pre‐defined, or clinically relevant and reasonably expected outcomes | Low. A matrix was created by review author and verified that there was a complete reporting of all pre‐defined, or clinically relevant and reasonably expected outcomes |
| **Other sources of bias** | Unsure. The study was supported by a grant from the European and Developing Countries Clinical Trials Partnership. Nuala Mc-Grath was supported by a Wellcome Trust Fellowship and Neal Alexander received support from the United Kingdom Medical Research Council and Department for Interna-tional Development | Unsure. The study was supported by the National Institute for Health Research Imperial Biomedical Research Centre and Imperial Clinical Trials Unit based at Imperial College Healthcare NHS Trust and Imperial College London | Unsure. The University of Utah Annette Poulson Cumming College of Nursing provided funding for this research. | Unsure. Dr. Mohr is supported by grants from the Emergency Medicine Foundation and the U.S. Department of Health and Human Services Health Resources and Services Administration. This study was supported by the University of Iowa Department of Emergency Medicine and the University of Iowa Carver College of Medicine (NIH training grant 2T35HL007485-36). The University of Iowa Department of Emergency Medicine sponsors a rural emergency telemedicine network | Low. | Low. |

**CASP Checklist for Cohort and Case control with reasons**

**Case Control**

|  | **Barrera-2016**^50^ | **Haussen-2017**^39^ | **Dobscha-2005**^42^ | **Lurie-2011**^43^ | **Swain-2017**^44^ |
| --- | --- | --- | --- | --- | --- |
| **Section A: Are the results of the trial valid?** | | | | | |
| **1. Did the study address a clearly focused issue?** | Yes. Given the relatively limited self-help online resources available to perinatal women, especially from developing nations, and the multiple vulnerabilities associated with this population (e.g., pregnancy-related symptoms of depression), this study examined whether participants would misunderstand the information detailed in the informed consent of a randomized controlled trial (RCT) | Yes. The trial aimed to describe the first experience with electronic informed consenting in trials of cerebral thrombectomy for patients with acute ischemic stroke | Yes. The study aimed to describe the effects of using videoconferencing on participant enrollment, research measure administration and responses, study retention, and satisfaction on a randomized clinical trial of a care management intervention for depression. | Yes. The objective of the trial was to evaluate the changes in treatment preference before and after watching a video decision aid as part of an informed consent process for spine surgery. | Yes. This pilot study set out to determine the impact of a 15-min culturally tailored educational video on three outcomes relating to clinical trials: likely participation, attitudes (assessed based on six barriers), and actual enrollment of black breast cancer patients. |
| **2. Did the authors use an appropriate method to answer their question?** | Can´t tell. Data for this report were extracted from the baseline assessment of a larger trial that examined the efficacy of an online prevention of postpartum depression (PPD) intervention (see Barrera, Wickham, & Muñoz, 2015). Participants were recruited online using sponsored links (see Barrera, Kelman, & Muñoz, 2014) and were directed to the online consent form if they were female, pregnant (at any stage), and over 18 years of age. | Can´t tell. This was a retrospective analysis of prospectively collected data with the use of e-Consent in thrombectomy trials at an institution. | Can´t tell. Patients recruited from distant clinic sites had the option of traveling for face to face interview or being interviewed using videoconferencing. Interviews included obtaining informed consent and administration of research measures. Remote participants were subsequently asked to complete a 12-item mail survey regarding the interview. | Can´t tell. Subjects enrolling in the Spine Patient Outcomes Research Trial (SPORT) with intervertebral disc herniation (IDH), spinal stenosis (SPS), or degenerative spondylolisthesis (DS) at thirteen multidisciplinary spine centers across the US were given an evidence-based videotape decision aid viewed prior to enrollment as part of informed consent. | Can´t tell. The primary aim of the current study was to explore the ability of the short video to impact black breast cancer patients’ actual participation in a therapeutic CT. Our secondary aims were to explore the capacity of the video to influence black breast cancer patients’ (a) intentions to participate in a CT; and, (b) attitudes towards CTs. Changes in (a) and (b) were measured using the The Attitudes and Intention to Enroll in Therapeutic Clinical Trials (AIET) questionnaire (Supplementary Table 1) administered at three separate time points: pre-video, immediately following the video (post-test), and between 7 and 21 days post-video intervention (follow-up). |
| **Is it worth continuing?** | | | | | |
| **3. Were the cases recruited in an acceptable way?** | Can´t tell. Participants were recruited online using sponsored links (review author can´t confirm if system is reliable) and were directed to the online consent form if they were female, pregnant (at any stage), and over 18 years of age. To ensure recruitment of the target population, interest in using the study materials for personal use was also part of the eligibility criteria. Participants were recruited from 5 different countries. Cases seem representative of a defined population. No power calculation was stated. | No. After evaluation of inclusion/exclusion criteria, investigators sought out the appropriate LAR.As soon as the LAR was reached, a discussion on the trial was performed by one physician/investigator via phone. Regardless of the geographical position of the LAR, a standard link to the URL is sent via text message and/or email to the LAR, generating a primary instrument (e-Consent form). There were few subjects recruited. There was no power calculation. | Can´t tell if participants were representative of a population. Between July 2002 and October 2003, 400 patients from the Portland Veterans Affairs Medical Center primary care clinics for DEP-PC were enrolled. Participants were identified for DEP-PC using computerized lists of patients who were due to see primary care providers within a month, and whose primary care providers were participating in DEPPC. No power calculation was stated. | Can´t tell. There was no specific recruitment detailed for this study. All patients were given an evidence-based video decision aid as part of their informed consent process; there were two separate video decision aids, one for the IDH group and another for the SPS and DS groups. A small percentage of the patients chose not to watch the video. After enrollment patient preferences (including those of non-watchers) were again assessed using the same scale | Can´t tell. Recruitment process was not sufficiently detailed in the report (Eligible participants were identified through review of electronic clinical schedules, patient medical records, tumor boards or physicians, nurse navigators, clinical research coordinators and support services staff referrals at the following hospital sites: WCI at MedStar Washington Hospital Center; Lombardi Comprehensive Cancer Center at MedStar Georgetown University Hospital; MedStar Union Memorial Hospital; MedStar Harbor Hospital; and MedStar Franklin Square Medical Center). |
| **4. Were the controls selected in an acceptable way?** | No. No controls were reported. | No. No controls were reported. | No. No controls were clearly reported. We would assume controls are the subjects who chose not to watch the video. | No. No controls were clearly reported. We would assume controls are the subjects who performed on-site visits but this is not stated in the report. | No. No controls were reported. |
| **5. Was the exposure accurately measured to minimise bias?** | Can´t tell. Questions in survey appeared to be objective measure but as survey was not included in the report, this coud not be confirmed. There is no reference to confirm that survey was validated. Study did not incorporate blinding. The exposure preceds the outcome. | Yes. Authors used objective measures (authors developed a novel e-Consent tool. REDCap (Research Electronic Data Capture; Vanderbilt University, TN) is a secure, Health Insurance Portability and Accountability Act compliant, web-based platform designed for research data capture. Data were collected and managed using the REDCap tools hosted at Emory University, deemed to provide an intuitive interface for validated data entry.2 An institutional review board application was approved. REDCap was used to create a survey project located on a static URL that can be remotely accessed (via smartphone or computer web browser)) | Can´t tell. Report does not clarify if survey was validated: (the questionnaire was adapted from the VA Telemedicine Patient Feedback form,11 which was developed to assess satisfaction of veterans who receive clinical care using videoconferencing. The questions were stated in both positive (agreement represents satisfaction) and negative terms (disagreement represents satisfaction), in order to reduce response bias. Response options were: strongly disagree, disagree, neutral, agree, and strongly agree). | Can´t tell. Report does not state if videos were validated (these videos were adapted for this trial from decision aids developed by and available through the Foundation for Informed Medical Decision Making. Each decision aid included basic information on the condition itself, testimonials by patients who had the condition and had chosen each of the options, and a summary of the available data on outcomes and potential harms of each of the treatment options derived from the literature). | Can´t tell. Report does not clarify if video and questionnaires were validated |
| **6. (a) Aside from the experimental intervention, were the groups treated equally?** | No. No controls were reported. | No. No controls were reported. | No. No controls were reported. | No. No controls were clearly reported. | No. No controls were reported. |
| **6. (b) Have the authors taken account of the potential confounding factors in the design and/or in their analysis?** | No. Not stated. | No. Not stated. | No. Not stated. | No. Not stated. | No. Not stated. |
| **Section B: What are the results?** | | | | | |
| **7. How large was the treatment effect?** | Can´t tell. T-test and chi-square analyses were used to compare group differences in understanding of consent query items based on MDE and randomization status. All data were analyzed using SPSS for Windows 20.0. Results were not adjusted for confounding. | Can´t tell. Enrolled e-Consented patients (n=4) had similar age (73±14 versus 69±12 years; P=0.65) and National Institutes of Health Stroke Scale (16±5 versus 16±5; P=0.88) as compared with conventionally consented (n=25). Time from door-to-randomization was decreased with e-Consenting (28±9 versus 57±24 minutes; P=0.002). | Can´t tell. Report states that Pearson product moment correlations and t tests were used to compare scores for patients interviewed by videoconferencing with DEP-PC patients interviewed in-person, using SPSS® Version 10.1 for Windows (SPSS Inc., Chicago, IL) and for all significance tests, two-tailed tests, and at 0.05 was used but results were not included in the report. | Can´t tell. Of 2505 patients, 86% (n=2151) watched the video and 14% (n=354) did not. Watchers shifted their preference more often than non-watchers(37.9% vs. 20.8%, p < 0.0001) and more often demonstrated a strengthened preference (26.2% vs. 11.1%, p < 0.0001). Among the 806 patients whose preference shifted after watching the video, 55% shifted toward surgery (p=0.003). | Can´t tell. Basic descriptive statistics were used to assess the distribution of sociodemographic and clinical characteristics in the study population. Bivariate associations between enrollment status and patient-level characteristics were examined by t-tests for continuous variables and chi-square and Fisher exact tests for categorical variables. McNemar and symmetry tests were used to examine changes in the responses to the attitudinal barriers and intention to enroll as a result of the video intervention. Given that every participant would be exposed to the intervention, logistic regression models were used to examine whether changes in attitudes (from pre-topost- intervention and upon follow-up) significantly influenced the actual decision to participate in a CT. Multiple imputations were used when necessary for missing data. Two-tailed p < .05 was considered statistically significant. Statistical analyses were performed using SAS, 9.4 (SAS Institute, Cary, NC). Results were not adjusted for confounding. |
| **8. How precise was the estimate of the treatment effect?** | Can´t tell. No p-value or confidence intervals reported as this was a qualitative research | Can´t tell. Confidence interval not reported and p-value is low | Can´t tell. No p-value or confidence interval reported | Can´t tell. Confidence interval not reported and p-value is low | Can´t tell. Confidence interval not reported and p-value is low |
| **9. Do you believe the results?** | Yes | Can´t tell. The small sample size in this study may have increased the risk of the research team being misled | Can´t tell. 369 patients interviewed in person and 31 interviewed by videoconference (20 of the 31 patients interviewed by videoconferencing returned the satisfaction questionnaire, representing a response rate of 64.5%). | Can´t tell. The major limitation of this study is that the videos were supplied to all subjects and there was no randomized comparison group that did not receive the decision aid | Can´t tell. Several potential study limitations are worthy of notation: (1) the non-randomized design that did not employ a control group; (2) the various stages of diagnosis and treatment at the time of participation may have impacted study participants’ attitudes toward therapeutic trials and intention/decision to enroll; (3) although men were not excluded from participating, our study represents only female patients’ perspectives; (4) a self-selection bias may exist, given the patients who agreed to participate in INSPIRE-BrC may have been likely to be less opposed to research in general; (5) participants were required to provide informed consent to take part in the study, which could have potentially influenced their responses to attitudinal measures on the AIET regarding consent; (6) there was no requirement that a trial was available at the time of participation, so at any given timepoint, a trial may not have been offered due to lack of trial availability for that given patient; and, (7) the overall sample size was small, providing limited power to detect nuanced differences in attitudes among this study population. |
| **Section C: Will the results help locally?** | | | | | |
| **10. Can the results be applied to the local population?** | Yes | Yes | Yes | Yes | Yes |
| **11. Do the results of this study fit with other available evidence?** | Can´t tell | Can´t tell | Can´t tell | Can´t tell. | Can´t tell |

**Cohort**

|  | **Ditai-2018**^48^ |
| --- | --- |
| **1. Did the study address a clearly focused issue?** | Yes. The effectiveness of audio-visual interventions adhering to Consolidated Standards of Reporting Trials (CONSORT), has not been conducted in underserved populations in low- and middleincome countries or with people with low literacy. In this study, we developed audio-visual presentations of the participant information to improve the informed consent process for women of low or no literacy in a low-income setting in rural eastern Uganda |
| **2. Was the cohort recruited in an acceptable way?** | No. Cohort was representative of study population. The study setting was homes of pregnant women from villages in Mbale District in rural eastern Uganda. In this study area, only 6% of women aged 15–49 years had completed education at secondary level or higher, compared to the national average of 33%. Literacy was less than 68%. Participants were not allocated at random to the three methods. Hence, participants in the three groups was not ensured to be as similar as possible to each other before the interventions were started. |
| **Is it worth continuing?** |  |
| **3. Was the exposure accurately measured to minimise bias?** | Yes. Objective procedures were used. Procedures reflect study objectives and report confirmed the system process by which they were developed (the participant information sheets, the slides in the flip charts and the videos were developed in a rigorous systematic stepwise process. The lead investigator trained the research assistants on the presentation of the three consent methods using role playing, peer reviews and critiques before participants were enrolled using any of the methods). |
| **4. Was the outcome accurately measured to minimise bias?** | Can´t tell. Objective measures were used. Measurements reflect study objectives but report does not confirm that tool was validated (quantitative data were collected electronically into the modified version of the Quality of Informed Consent (QuIC) case report form on the ODK system operated smartphones. The form or data tool was designed to measure objective and subjective comprehension of the study information). Qualitative data was collected through semi-structured interviews, two qualitative researchers explored aspects of the comprehension of the informed consent messages and the preferences relating to the informed consent process. All responses were audio-recorded and the qualitative researchers entered notes directly into the ODK software on the smartphone. Same procedure was used in all subjects. |
| **5. (a) Have the authors identified all important confounding factors?** | No. No confounding factors were identified. |
| **5. (b) Have they taken account of the confounding factors in the design and/or analysis?** | No. No confounding factors were identified. |
| **6. (a) Was the follow up of subjects complete enough?** | No. All subjects were followed up at 48hs as stated in outcomes. There were no lost to follow up subjects. Participants’ recall of the information was measured only within 48 h and their recall and understanding after a long time, especially in the long-term follow-up during a trial, may be different. |
| **7. What are the results of this study?** | Yes. A total of 30 pregnant women in their homes participated in this study. Their recall of the trial information within the planned 48 h was assessed for the majority (90%, 27/30). For all three consent models, women demonstrated a high understanding of the study. There was no statistically significant difference between the slide-show message (mean 4.7; standard deviation, SD 0.47; range 4–5), video message (mean 4.9; SD 0.33; range 4–5) and standard method (mean 4.5; SD 0.53; range 4–5; all one-way ANOVA, p = 0.190). The slide-show message resulted in the most objective understanding of question items with the highest average QuIC score of 100 points. For women who had been recruited using any of the three models, the slide show was the most popular method, with a mean score for all items of not less than 4.2 (mean 4.8; SD 0.6; range 4–5). Most women (63%, 19/30) preferred the slide-show message, compared with 17% (5/30) and 20% (6/30) for the standard and video messages, respectively. |
| **8. How precise are the results?** | Yes. Confidence intervals are relatively narrow. |
| **9. Do you believe the results?** | Can´t tell. The small sample size in this study may have increased the risk of the research team being misled by chance differences in the three consent methods. Larger studies would provide more statistical power to explore the factors that improve understanding. |
| **10. Can the results be applied to the local population?** | No. Subjects covered in this study are specific and different from other populations |
| **11. Do the results of this study fit with other available evidence?** | Can´t tell. this is the first study to compare video and illustrated participant trial information with the long-established researcher-read method in a rural African setting. |
| **12. What are the implications of this study for practice?** | Can´t tell. Implication for practice were not reported. Only mentioned that appropriate interactions and communications between participants and researchers are vital for improving participants’ understanding of the informed consent process |
